# How the Way a Naphthalimide Unit is Implemented Affects the Photophysical and -catalytic Properties of Cu(I) Photosensitizers

**DOI:** 10.3389/fchem.2022.936863

**Published:** 2022-06-17

**Authors:** Yingya Yang, Florian Doettinger, Christian Kleeberg, Wolfgang Frey, Michael Karnahl, Stefanie Tschierlei

**Affiliations:** ^1^ TU Braunschweig, Institute of Physical and Theoretical Chemistry, Department of Energy Conversion, Braunschweig, Germany; ^2^ TU Braunschweig, Institute of Inorganic and Analytical Chemistry, Braunschweig, Germany; ^3^ University of Stuttgart, Institute of Organic Chemistry, Stuttgart, Germany

**Keywords:** copper photosensitizer, X-ray structures, time-resolved spectroscopy, excited-state properties, DFT calculations, singlet oxygen

## Abstract

Driven by the great potential of solar energy conversion this study comprises the evaluation and comparison of two different design approaches for the improvement of copper based photosensitizers. In particular, the distinction between the effects of a covalently linked and a directly fused naphthalimide unit was assessed. For this purpose, the two heteroleptic Cu(I) complexes **CuNIphen** (**NIphen** = 5-(1,8-naphthalimide)-1,10-phenanthroline) and **Cubiipo** (**biipo** = 16H-benzo-[4′,5′]-isoquinolino-[2′,1′,:1,2]-imidazo-[4,5-f]-[1,10]-phenanthroline-16-one) were prepared and compared with the novel unsubstituted reference compound **Cuphen** (**phen** = 1,10-phenanthroline). Beside a comprehensive structural characterization, including two-dimensional nuclear magnetic resonance spectroscopy and X-ray analysis, a combination of electrochemistry, steady-state and time-resolved spectroscopy was used to determine the electrochemical and photophysical properties in detail. The nature of the excited states was further examined by (time-dependent) density functional theory (TD-DFT) calculations. It was found that **CuNIphen** exhibits a greatly enhanced absorption in the visible and a strong dependency of the excited state lifetimes on the chosen solvent. For example, the lifetime of **CuNIphen** extends from 0.37 µs in CH_2_Cl_2_ to 19.24 µs in MeCN, while it decreases from 128.39 to 2.6 µs in **Cubiipo**. Furthermore, **CuNIphen** has an exceptional photostability, allowing for an efficient and repetitive production of singlet oxygen with quantum yields of about 32%.

## Graphical Abstract

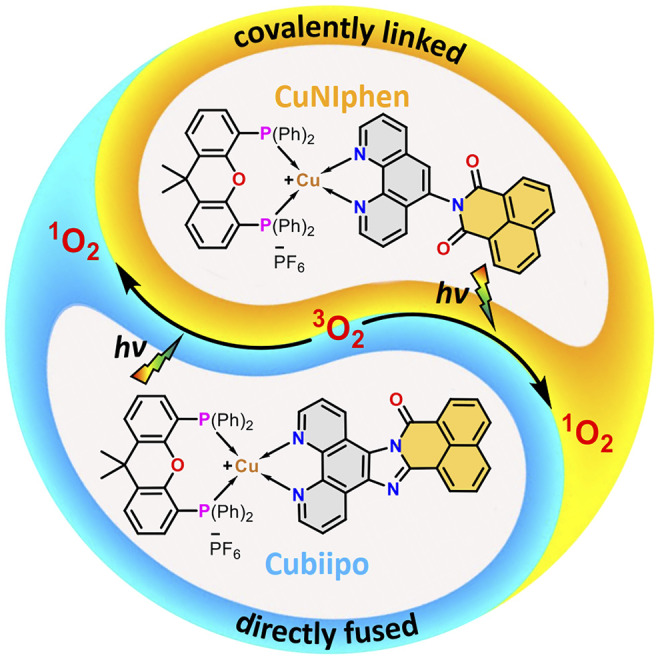

## Introduction

The motivation to resolve the conflict between the world’s increasing energy demand and the depletion of fossil fuels drives the search for more environmentally friendly and renewable energy sources (Detz et al., 2018; Gür, 2018; Stephen Nalley, 2021). One of the most attractive alternatives is the enhanced use of solar energy, which can be realized by its conversion and storage in electric energy and different chemical forms (Armaroli and Balzani, 2016; Hammarström, 2016; Lewis, 2016).

In this context, photosensitizers play an essential role, as they are able to harvest the sunlight to subsequently drive various photocatalytic reactions (Schultz and Yoon, 2014; Lewis, 2016; Capaldo and Ravelli, 2020). One the one hand, the high photostability and non-toxicity of semiconductors and, on the other hand, the wide absorption range and low costs of organic dyes attracted great research interests for decades (Lang et al., 2014; Nalzala Thomas et al., 2021). However, the rapid recombination of photogenerated electron-hole pairs and the resulting low photoefficiency of semiconductors as well as the low photostability of organic dyes limit their applicability (Cao et al., 2015; Penu et al., 2015; Chen et al., 2018; Nalzala Thomas et al., 2021; Dong et al., 2022). Therefore, due to the tunable redox and excited state properties of transition-metal complexes, there is an intensive search for efficient and robust transition-metal based photosensitizers (Eckenhoff and Eisenberg, 2012; Frischmann et al., 2013; Berardi et al., 2014; Yuan et al., 2017). Owing to their long-lived excited states, high absorption coefficients in the visible region and intense luminescence, mainly systems containing noble and expensive 4d/5d metals like Pt (Schultz and Yoon, 2014; Teegardin et al., 2016), Ru (Prier et al., 2013; Hagfeldt et al., 2010), Ir (Chi and Chou, 2010; Yuan et al., 2017) or Re (Yarnell et al., 2011; Wells et al., 2021) have been extensively studied.

In recent years, more cost-efficient and noble-metal-free 3d systems, based on for example Cr (Büldt and Wenger, 2017; Treiling et al., 2019; Reichenauer et al., 2021), Fe (Wenger, 2019; Obermeier et al., 2021; Leis et al., 2022) or Cu (Gernert et al., 2020; Schulz et al., 2020; Forero Cortés et al., 2021; Wegeberg and Wenger, 2021), have attracted increasing attention as suitable alternatives to rare metal complexes (Zhang et al., 2018a; Forero Cortés et al., 2021). In particular, heteroleptic copper (I) complexes with the general formula [(P^∧^P)Cu(N^∧^N)]^+^ bearing a diphosphine and a diimine ligand have been explored well due to their advantageous properties, such as very long-lived excited states (Armaroli, 2001; Kuang et al., 2002; Hagfeldt et al., 2010; Lazorski and Castellano, 2014; Paria and Reiser, 2014; Tsubomura et al., 2015; Heberle et al., 2017; Leoni et al., 2018; Zhang et al., 2018b). As a result, these complexes succeeded in a wide range of applications, including the photocatalytic production of hydrogen (Luo et al., 2013; Kim et al., 2017; McCullough et al., 2018), the activation of CO_2_ (Takeda et al., 2016; Call et al., 2019; Steinlechner et al., 2019; Giereth et al., 2021) or as dyes in organic light-emitting diodes (OLEDs) (Volz et al., 2013; Paria and Reiser, 2018; Au, 2021) and dye-sensitized solar cells (DSSCs) (Dragonetti et al., 2018; Dragonetti et al., 2019; Colombo et al., 2021).

As a consequence of their limited absorptivity in the visible region, numerous efforts have been made to modify and to adjust the diimine ligand. For example, several phenanthroline derivatives bearing phenyl and alkynyl substituents (Mejía et al., 2013; Chen et al., 2017; Kim et al., 2017; Zhang et al., 2018a; Doettinger et al., 2021; Forero Cortés et al., 2021) or (diaza)anthracene (Heberle et al., 2017; Soulis et al., 2018; Giereth et al., 2019) moieties were explored, but with limited success. Therefore, in some other studies naphthalene imides were applied as a promising alternative (Tyson et al., 2001; Yarnell et al., 2011; Castellano, 2015; Argüello Cordero et al., 2022). Further, the beneficial features of naphthalimide and diimide derivatives, such as great thermal and oxidative stability, high electron affinity and electron storage capability, are reasons for their widespread use as ligands or building blocks in bichromophoric systems (Castellano, 2015; Georgiev et al., 2016) and in different photocatalytic applications (Suraru and Würthner, 2014; Würthner et al., 2016; Reiß and Wagenknecht, 2019).

Attaching a naphthalimide moiety as an electron acceptor *via* covalent linkage (*i.e.*, by means of a single bond) to the backbone of an electron donor ligand like phenanthroline can impressively increase the extinction coefficients and excited state lifetimes of the corresponding complexes.(Tyson et al., 2001; Yarnell et al., 2011; Yarnell et al., 2019). As an alternative, recent studies revealed that a direct fusion (*e.g.*, *via* condensation reactions) of the naphthalene imide moiety to the phenanthroline backbone leads to a fully conjugated system. This resulted in more efficient light absorption far into the visible region and extended lifetimes of up to hundreds of microseconds in the solid state (Yang et al., 2020; Wells et al., 2021; Argüello Cordero et al., 2022).

**SCHEME 1:**
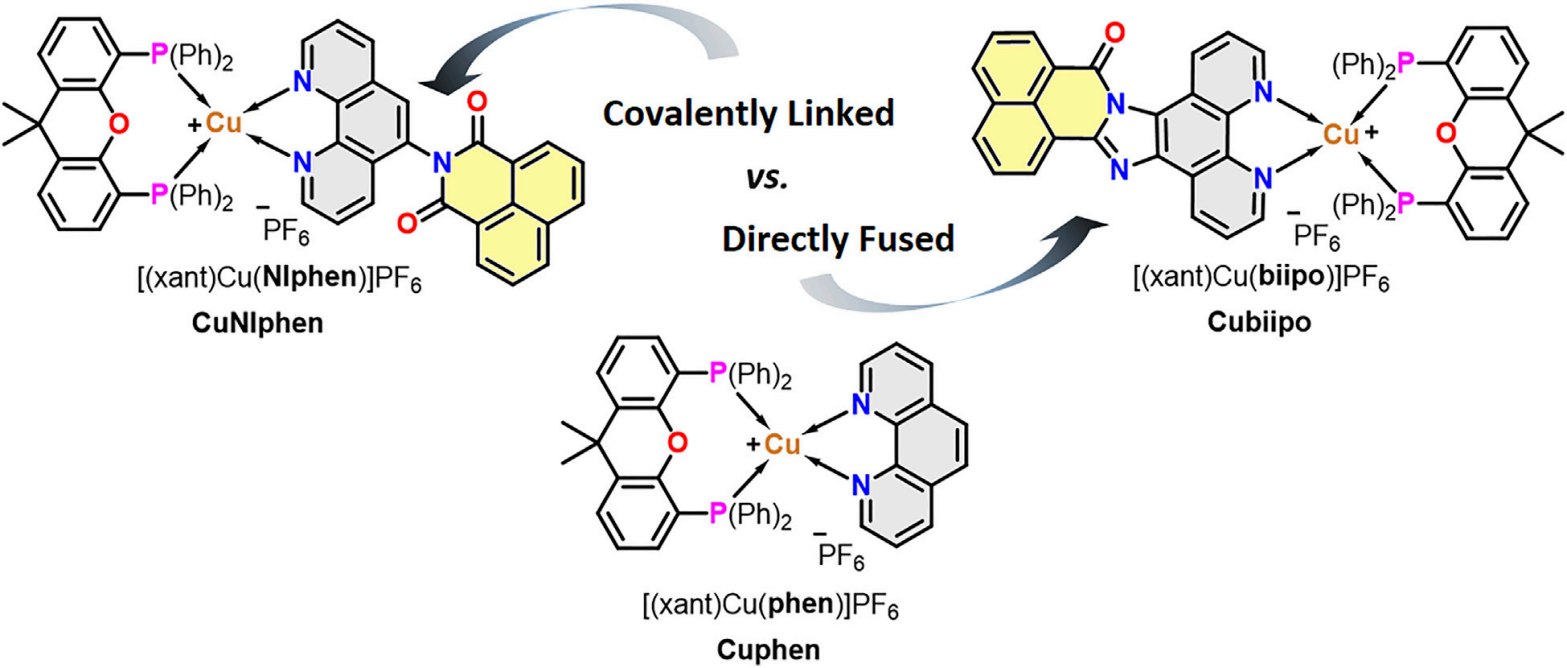
Overview of the chemical structures of the three Cu(I) photosensitizers and the two different design strategies (*i.e*. covalent linkage *vs.* directly fusing of a naphthalimide unit) compared in this study. **CuNIphen** and **Cuphen** are presented for the first time, whereas **Cubiipo** was taken from previous studies. (Yang et al., 2020; Argüello Cordero et al., 2022)

It is therefore of great interest to test and to compare these two approaches (*i.e.* covalent linkage *vs.* directly fusing) using the same type of metal complexes (Scheme 1) and to study the effects on the redox and photophysical behavior as well as on the photoactivity. To this end, we prepared two novel heteroleptic Cu(I) complexes, namely **CuNIphen** and **Cuphen**, which are based on the **NIphen** ligand bearing a covalently linked naphthalimide unit (**NIphen** = 5-(1,8-naphthalimide)-1,10-phenanthroline) and the mere **phen** ligand (= 1,10-phenanthroline), respectively (Scheme 1). By comparing these two complexes with the already known **Cubiipo**, (**biipo** = 16H-benzo-[4′,5′]-isoquinolino-[2′,1′,:1,2]-imidazo-[4,5-f]-[1,10]-phenanthrolin-16-one) we seek to explore how these three modifications affect the orbital energies of the diimine ligand, and thus, the ^3^LC and ^3^MLCT states, as well as the resulting change in redox behavior and charge transfer efficiency. To this end, two novel solid state structures (**CuNIphen** and **Cuphen**) are presented and various steady state and time-resolved spectroscopic techniques, electrochemical measurements as well as time-dependent density functional theory (TD-DFT) are applied. Moreover, the new complex was successfully tested over several cycles in the light-driven generation of singlet oxygen (^1^O_2_). The efficiency of the ^1^O_2_ generation was examined also considering its continuity and photostability.

## Experimental Details

The substituted phenanthroline ligand 5-(1,8-naphthalimide)-1,10-phenanthroline (**NIphen**) was synthesized following a literature known procedure.Yarnell et al. (2019) 1,10-Phenanthroline (**phen)** was purchased commercially. The **biipo** ligand and the corresponding [(xant)Cu(**biipo**)]PF_6_ complex (**Cubiipo**), where xant corresponds to the xantphos ligand, were required for comparison and taken from previous studies (Yang et al., 2020; Argüello Cordero et al., 2022).

The new heteroleptic copper (I) complexes **CuNIphen** and **Cuphen** were prepared *via* an one-pot, two-step approach, which is already known in the literature (Heberle et al., 2017; Rentschler et al., 2020). For that purpose, the xantphos ligand was first coordinated to the [Cu(MeCN)_4_]^+^ (MeCN = acetonitrile) precursor to form the [(xant)Cu(MeCN)_2_]^+^ intermediate in dichloromethane (CH_2_Cl_2_) solution at 40°C under inert conditions (Luo et al., 2013; Mejía et al., 2013). In a second step the respective diimine ligand (**NIphen** or **phen**) was coordinated by slow addition of 1 equivalent of the diimine in CH_2_Cl_2_ solution at 0°C under exclusion of oxygen. In order to increase the yield and purity while facilitating the synthetic efforts at the same time, the literature known procedure was slightly modified for **Cuphen** as follows: Instead of adding a CH_2_Cl_2_ solution of **phen** dropwise by hand *via* syringe, an automatic syringe pump was used to precisely control the volume flow rate (*i.e.*, 13 ml/h, for further information see Supplementary Material S2). This guarantees the exclusive formation of the heteroleptic target complex in the absence of the respective thermodynamically favored homoleptic bis-diimine complex (*e.g.*, [Cu(**phen**)_2_]^+^) (Kaeser et al., 2013; Fischer et al., 2014; Lennox et al., 2016) and eases the synthesis. After precipitation from the CH_2_Cl_2_ solution by carefully adding *n*-hexane, the final yields of the pure bright yellow target compounds were 38% for **CuNIphen** (after two additional recrystallization steps) and 80% for **Cuphen**.

## Results and Discussion

### Structural Characterization

Both complexes were fully characterized by nuclear magnetic resonance (NMR) spectroscopy (^1^H, ^13^C, ^31^P), high resolution mass spectrometry (HRMS) and elemental analysis (EA). **CuNIphen** was further investigated by 2D-NMR spectroscopy, namely H-H nuclear overhauser effect spectroscopy (H-H-NOESY) and heteronuclear multiple bond correlation (HMBC), which allowed accurate assignment of all proton signals (Supplementary Material S2 and S3). In addition, the solid-state structures of **CuNIphen** and **Cuphen** were determined by single crystal X-ray crystallography and are compared with molecular structures that resulted from density functional theory (DFT) calculations.

#### Nuclear Magnetic Resonance

The asymmetric substitution at the 5-position of the phenanthroline ligand causes two separated signals from the two methyl groups in the xantphos backbone at 1.78 ppm (*s*, 3H, CH_3_) and 1.74 ppm (*s*, 3H, CH_3_) in the ^1^H NMR spectrum of **CuNIphen (** Supplementary Figure S1). Similar observations were made for **Cubiipo** (Argüello Cordero et al., 2022) and other asymmetrically substituted [(xant)Cu(diimine)]^+^ complexes (Schmid et al., 2018). This in strong contrast to the commonly observed single signal at around 1.7–1.8 ppm for the two methyl groups (*s*, 6H, CH_3_) in related symmetrically substituted Cu(I) complexes (Luo et al., 2013; Mejía et al., 2013; Kim et al., 2017; Doettinger et al., 2021) and is also in contrast to the unsubstituted **Cuphen** complex (1.76 ppm, *s*, 6H, CH_3_) (Supplementary Figure S7). The same splitting phenomenon can also be seen in the ^13^C NMR spectra of **CuNIphen** and **Cubiipo**. There, the chemical shifts of both methyl groups are clearly separated (28.60 and 28.20 ppm for CuNIphen, 27.3 and 27.1 ppm for Cubiipo (Argüello Cordero et al., 2022)), whereas in **Cuphen** only one signal appears (28.55 ppm) (Supplementary Figures S4, S5, S8). By measuring their mutual interactions through space *via* NOESY, it has now been confirmed that these signals each correspond to one methyl group within the same xantphos ligand framework and are not caused by two different molecular species (Supplementary Figure S2). This is further supported by the HMBC spectrum, where both sets of protons are coupling to the carbon connecting the two methyl groups (Supplementary Figure S3).

**FIGURE 1:**
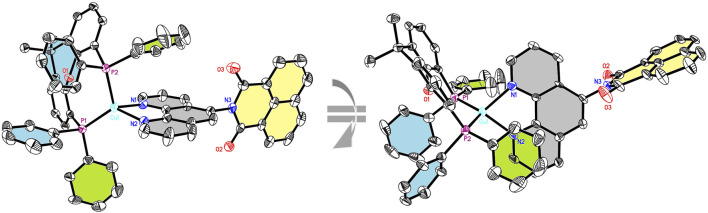
Solid-state structure (two different ORTEP representations) of **CuNIphen** with thermal ellipsoids at a probability level of 50%. Hydrogen atoms, counter anions, and solvent molecules are omitted for clarity. For comparison with **Cuphen**, Supplementary Figure S11.

The previously discussed findings are, however, no evidence that the two regioisomers do not occur in solution. The asymmetric substitution causes the signals of the *o*-protons of the phenyl groups on the phosphorus in **Cuphen** at 6.92 ppm to split into two different sets at 7.01 ppm (4H) and 6.90 ppm (4H) in **CuNIphen** — two closer (green) and two distal (blue) from the diimine ligand (*cf.* Figure 1). Both sets exhibit through-space interactions with the protons at the 2- and the 9-position of the phenanthroline (adjacent to N1 and N2 in Figure 1), which is only reasonable if two regioisomers are present (Supplementary Figure S26 for graphical explanation).

This strongly suggests that indeed both possible regioisomers are formed during complexation and occur in solution. Furthermore, it should be noted that during synthesis (where the diimine ligand is coordinated to the preformed intermediate complex) a differentiation between both possible orientations is unlikely. This conclusion is also supported by DFT calculations, as they predict only a small thermodynamic energy difference of 1.39 kJ/mol between both isomers (Supplementary Figure S26).

#### X-ray Characterization

To further analyze the structure of the new compounds, single crystals of **CuNIphen** and **Cuphen** suitable for X-ray crystallography were obtained by slow diffusion of *n*-heptane into a concentrated CH_2_Cl_2_ solution of the respective complex (Supplementary Material S3.3 for further details). **CuNIphen** (Figure 1) exhibits an intramolecular π-stacking interaction between one phenyl group (green) of the xantphos ligand and the phenanthroline ligand (grey). More precisely, the phenyl ring directly overlays the pyridine moiety opposite to the substituted 5-position due to steric effects (*cf.* Figure 1, right). Interestingly, a comparable π-stacking interaction is not present in **Cuphen**, although the unsubstituted 1,10-phenanthroline does not carry a sterically demanding substituent (Supplementary Figure S12). This is most probably due to competing intermolecular interactions in the solid state.

It should be mentioned, that for **CuNIphen** only one regioisomer was detected in the X-ray crystallographic measurements. In contrast, two regioisomers were found for **Cubiipo**, which also has an asymmetric structure (Argüello Cordero et al., 2022). However, the structural difference between the two possible regioisomers of **CuNIphen** is larger than for **Cubiipo**. This means that **Cubiipo** possesses a high overall planarity due to the **biipo** ligand, (Argüello Cordero et al., 2022) whereas in **CuNIphen** the torsion angle of the substituent is almost 90° as a result of the covalently linked naphthalimide (Table 1), inducing a greater steric bulk. It is therefore likely that one isomer is preferred during the crystallization process. DFT calculations (B3LYP-D3(BJ)/def2-TZVP) were used to predict the structures of **CuNIphen** and **Cuphen** (Supplementary Figures S26, S27) simulated in acetonitrile solution. For **CuNIphen** both possible regioisomers were evaluated and only a small thermodynamic energy difference of 1.39 kJ/mol was predicted, with the structure also observed in the solid state (Figure 1) being energetically favored. The structural parameters obtained from DFT were compared to those from X-ray studies and are in good agreement (Table 1).

**TABLE 1:** Selected bond lengths, bond angles and interplanar angles (N-Cu-N *vs*. P-Cu-P) of **Cuphen** (top) and **CuNIphen** (bottom) including the substituent torsion angle (phen *vs*. naphthalimide). The interplanar angle corresponds to the angle between the two N-Cu-N and P-Cu-P planes, respectively. The bond length C*-N3 describes C-N bond from the phenanthroline to the naphthalimide. The table also compares the experimentally determined parameters (exp.) with those calculated by DFT (denoted italicized as *calc.*). Further information can be found in the Supplementary Material S3.3).

**Cuphen**								
Selected bond lengths				Selected bond angles				
Atom 1	Atom 2	Length (pm): exp. *vs. calc.*		Atom 1	Atom 2	Atom 3	Angle (°): exp. *vs. calc.*	
Cu	P1	228.49 (5)	*229.5*	P1	Cu	P2	114.72 (2)	*118.0*
Cu	P2	224.36 (6)	*223.5*	N1	Cu	N2	80.26 (5)	*80.2*
Cu	N1	205.1 (1)	*210.7*	Interplanar angle (°): exp. *vs. calc.*				
Cu	N2	213.0 (1)	*210.8*			77.36	*87.63*	
**CuNIphen**								
**Selected bond lengths**				**Selected bond angles**				
Atom 1	Atom 2	Length (pm): exp. *vs. calc.*		Atom 1	Atom 2	Atom 3	Angle (°): exp. *vs. calc.*	
Cu	P1	224.7 (2)	*223.8*	P1	Cu	P2	117.90 (6)	*118.1*
Cu	P2	230.2 (2)	*229.3*	N1	Cu	N2	80.4 (2)	*79.9*
Cu	N1	209.8 (5)	*212.1*	Interplanar angle (°): exp. *vs. calc.*				
Cu	N2	206.4 (5)	*209.9*			82.15	*86.70*	
C*	N3	144.8 (7)	*143.9*	Substituent torsion angle (°): exp. *vs. calc.*				
						88.97	*88.24*	

Concerning the geometry proximal to the copper center, it can be deduced from Table 1 and Figure 1 that **Cuphen** and **CuNIphen** both exhibit a distorted tetrahedral geometry (described as *interplanar angles*) which is common for this class of copper complexes (Leoni et al., 2018; Keller et al., 2020; Doettinger et al., 2021) including **Cubiipo** (Argüello Cordero et al., 2022). The bond angles (*e.g.*, P1-Cu-P2 and N1-Cu-N2) and bond lengths (Cu-P1 and Cu-N1) are very similar to each other and are in the same dimension as described earlier. Further, also the bond length between the carbon and the nitrogen atoms linking the naphthalimide substituent (C*-N3 = 144.8 p.m., Table 1) is in good agreement with a comparable structure in the literature (144.4 p.m. (Zhang and Ma, 2019). This renders both complexes **CuNIphen** and **Cuphen** as structurally closely related compared to similar Cu(I) compounds.

### Electrochemical Properties

**FIGURE 2:**
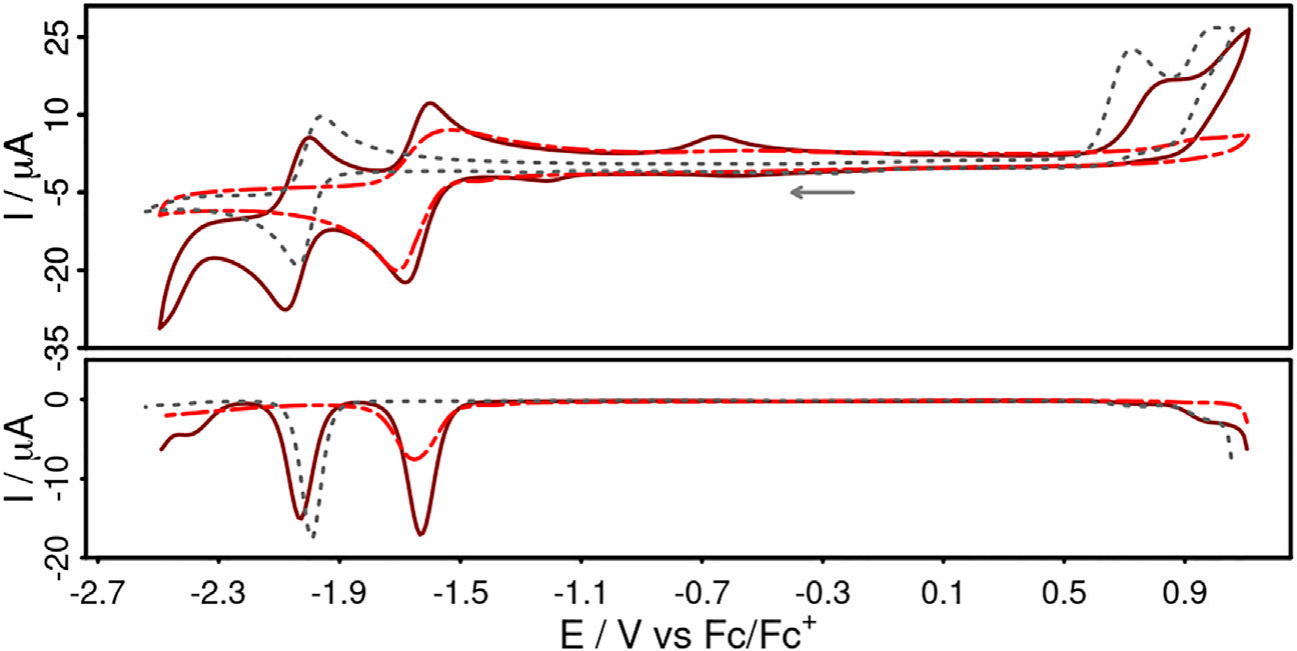
Cyclic voltammograms (top) and differential pulse voltammograms (bottom) of **CuNIphen** (dark red, solid, 1 mM), **NIphen** (red, dashed, 1 mM) and **Cuphen** (grey, dotted, 1 mM) in acetonitrile solution referenced *vs.* the ferrocene/ferricenium (Fc/Fc^+^) couple. Conditions: scan rate of 100 mVs^−1^, [Bu_4_N][PF_6_] (0.1 M) as supporting electrolyte.

The electrochemical properties of **Cuphen**, **CuNIphen** and the corresponding **NIphen** ligand were determined by cyclic and differential pulse voltammetry (Figure 2) in acetonitrile solution containing 0.1 M [Bu_4_N][PF_6_] as electrolyte. The electrochemical data of **Cubiipo** and **biipo** are also given for comparison and are summarized in Table 2.

**TABLE 2:** | Summary of the photophysical and electrochemical properties of **NIphen**, **CuNIphen**, **Cuphen** and of selected reference compounds in acetonitrile and dichloromethane solution at room temperature. The absorption measurements were carried out in MeCN solution. The electrochemical data were obtained from deaerated acetonitrile solution at room temperature and are referenced *vs.* the ferrocene/ferricenium (Fc/Fc^+^) couple.

Compound	$\lambda_{\mathrm{abs}}$ [nm] ( ε [10^3^ M^−1^ cm^−1^])	$\lambda_{\mathrm{em}}$ [nm]		*τ*_excited state_ [µs]		*τ*_em_ [ns]	$\phi_{1_{O_{2}}}$	$E_{\frac{1}{2}\mathrm{ox}}$ [V]	$E_{\frac{1}{2}\mathrm{red}}$ [V]
		MeCN	CH_2_Cl_2_	MeCN	CH_2_Cl_2_	MeCN	CH_2_Cl_2_		
**NIphen**	334 (9.9)	375^b^ ^,^ ^c^	377^b^ ^,^ ^c^	8.40^a^ ^,^ ^c^	11.04^a^ ^,^ ^c^	*n.d.*	*n.d.*	-	- 1.61^l^
**CuNIphen**	385 (4.8)	-	654^b^ ^,^ ^d^	0.42^b^ ^,^ ^c^	0.37^a^ ^,^ ^c^ ^,^ ^f^	<10^g^	0.32^b^ ^,^ ^d^ ^,^ ^j^	+0.82^k^	-1.62^l^, -2.01^l^
			669^a^ ^,^ ^e^	19.24^a^ ^,^ ^c^ ^,^ ^f^					
**Cuphen**	380 (3.0)	-	654^b^ ^,^ ^d^	-	0.50^a^ ^,^ ^c^ ^,^ ^f^	<10 ^g^	0.38^b^ ^,^ ^d^ ^,^ ^j^	+0.72^k^	-2.00^l^
			661^a^ ^,^ ^d^ ^,^ ^e^						
**biipo**	409 (6.2)^h^	557^i^	*n.d.*	*n.d.*	*n.d.*	9.4^i^	*n.d.*	-	- 1.59^h^ ^,^ ^l^
**Cubiipo**	406 (16.3)^i^	556^i^ ^,^ ^m^	494^b^ ^,^ ^d^ ^,^ ^m^	0.27^b^ ^,^ ^i^ 2.6^a^ ^,^ ^i^	128.39^a^ ^,^ ^c^ ^,^ ^f^	9.7^i^	0.98^b^ ^,^ ^d^ ^,^ ^j^	+0.72^i^ ^,^ ^k^	-1.49^i^ ^,^ ^l^ -2.17^i^ ^,^ ^l^

a Under argon atmosphere.

b Under aerated conditions.

c Excitation wavelength of 355 nm.

d Excited at 387 nm.

e Excitation wavelength of 400 nm.

f Average time constant determined from single wavelength kinetic analysis.

g The lifetime was below the detection limit.

h Value taken from reference (Yang et al., 2020).

i Value taken from reference (Argüello Cordero et al., 2022).

j Using phenalenone ( $\phi_{1_{{\text{O}}_{2}}}=0.98$ ) in CH_2_Cl_2_ as reference (Epelde-Elezcano et al., 2016), (Gallavardin et al., 2012).

k Irreversible.

l Reversible.

m Weak emission originating from dissociated ligand. The absorption and electrochemical measurements were carried out in MeCN. - = below detection limit/no signal. n.d. = not determined.

**CuNIphen** features two reversible reduction waves at −1.62 V and −2.01 V and an irreversible oxidation wave at 0.82 V (dark red, solid). In contrast, **Cuphen** shows only one reversible reduction at −2.00 V and one irreversible oxidation wave at 0.72 V (grey, dotted). Both oxidation waves are assigned to the Cu(I)/Cu(II) oxidation and the literature-known cleavage of the Cu-P bond (Zhang et al., 2019; Forero Cortés et al., 2021). The **NIphen** ligand possesses one reversible reduction event at -1.62 V (red, dashed). Hence, the reversible reduction events of **CuNIphen** can be assigned to the stepwise reduction of 1) the naphthalimide substituent at -1.62 V and 2) the phenanthroline moiety at −2.01 V. This means that, analogous to **Cubiipo** (Yang et al., 2020; Argüello Cordero et al., 2022), **CuNIphen** can also be reduced twice and that the covalent linkage of the naphthalimide moiety to the phenanthroline core enables an additional second reduction. The fact that the first reduction event occurs at the distal substituent could also be confirmed by DFT calculations of the spin density of the singly reduced species of **CuNIphen** ([(xant)Cu(NIphen)]^±0^). Identical calculations for **Cuphen** predicted an increase in electron density at the phenanthroline, which is also in accordance with the experiment (Supplementary Figure S34 for spin density plots and further details).

It should be noted that the differences in the first reduction between the **NIphen** ligand and the respective Cu(I) complex are very small. In strong contrast, much larger potential differences (∆ = 100 mV) were observed between **biipo** and **Cubiipo** (Table 2). This indicates a more pronounced electronic communication between the phenanthroline moiety and the directly fused naphthalimide unit within the **biipo** system. For the second reduction a much stronger anodic shift was observed for **Cubiipo** (−2.17 V) compared to **CuNIphen** (−2.01V). Hence, the twofold reduction of the **CuNIphen** complex is much easier to perform than of **Cubiipo** (∆ = 160 mV). All in all, the covalent linkage of a naphthalimide substituent leads to a weaker electronic interaction between the phenanthroline and the naphthalimide moiety, but facilitates access to the doubly reduced species.

### Absorption and Steady State Emission Spectroscopy

**FIGURE 3:**
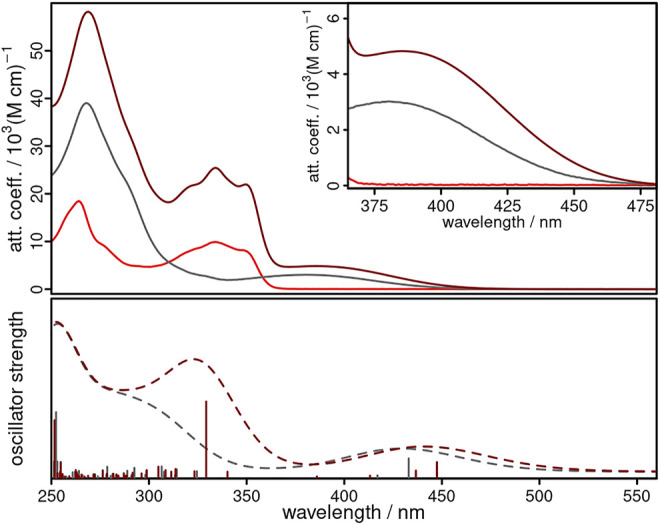
Experimental (top, solid lines) and calculated (bottom, dashed lines, B3LYP-D3(BJ)/def2-tzvp) UV/vis absorption spectra of **NIphen** (light red), **CuNIphen** (dark red) and **Cuphen** (grey) in acetonitrile. The inset shows an enlarged region of the absorption from 365 to 480 nm.

The UV/vis absorption spectra of **CuNIphen**, **NIphen**, and **Cuphen** show strong features between 250 and 300 nm in acetonitrile solution (Figure 3 and Table 2) originating from ligand centered (LC) π-π* transitions within the phenanthroline ligand, which is also in agreement with the literature (Accorsi et al., 2009; Zhang et al., 2016). The low-energy absorption bands of **NIphen** between 311 and 365 nm are predominantly due to LC transitions inside the naphthalimide unit (Xiao et al., 2010; Szakács et al., 2019; Yarnell et al., 2019). **Cuphen** has a broad absorption band centered at 380 nm, which is assigned *via* TD-DFT calculations to d_Cu_→π∗_phen_ metal-to-ligand charge transfer (MLCT) transitions (Supplementary Figure S29 and Supplementary Table S5). Interestingly, the spectrum of **CuNIphen** agrees well with the sum of the spectra of **NIphen** and **Cuphen**. This confirms the conclusion from the electrochemical measurements, that the electronic communication between both building blocks (*i.e*., naphthalimide and phenanthroline) is comparatively small. Nevertheless, **CuNIphen** possesses remarkably increased attenuation coefficients compared to the pure **NIphen** ligand and the reference compound **Cuphen** (*cf*. grey and red spectrum in Figure 3) in both the UV and especially the important visible range.

In addition, the UV/vis absorption spectra of **NIphen** and **CuNIphen** exhibit quite different features when compared to those of **biipo** and **Cubiipo**. In the **biipo** ligand, ligand centered (LC) π-π* transitions from the phenanthroline/imidazole part to the naphthaloylenebenzene moiety were observed in the 360–460 nm range (Yang et al., 2020). The MLCT transitions of **Cubiipo** overlap with these, resulting in a broad absorption band and high attenuation coefficients (Argüello Cordero et al., 2022). In contrast, the decreased electronic interaction in the covalently linked **CuNIphen** complex leads to two separated absorption areas at about 310–370 nm (the LC region) and at 370–470 nm (the MLCT region), and thus, lower attenuation coefficients. As a direct consequence of the missing overlap, **CuNIphen** generally absorbs weaker in the visible range than **Cubiipo** (Table 2).

In a next step, the steady state emission was studied, where **NIphen** exhibits a similar behavior in MeCN and CH_2_Cl_2_ solution with emission quantum yields of 0.057 and 0.034, respectively (Supplementary Figure S17). The moderate emission intensity of **NIphen** in both solvents indicates efficient intersystem crossing (ISC) from a bright ^1^LC to a dark ^3^LC state (Yarnell et al., 2019). In contrast to **NIphen**, the emission performance of **CuNIphen** is strongly solvent dependent. No emission of **CuNIphen** was observed in MeCN under inert conditions, whereas significant emission was found in deaerated CH_2_Cl_2_ with a maximum at 669 nm. Similarly, **Cuphen** displays no emission in MeCN, but a bright emission in CH_2_Cl_2_. In contrast, **Cubiipo** is not emissive in both solvents as previously studied. However, some emission is detected in solution due to uncoordinated ligand (Argüello Cordero et al., 2022).

### Excited State Properties

The excited states characteristics of **NIphen**, **CuNIphen**, **Cuphen**, and **Cubiipo** were further studied by time-resolved emission and transient absorption (TA) spectroscopy with an excitation at 355 nm in MeCN and CH_2_Cl_2_ (Supplementary Material S6).

The emission lifetimes of **NIphen** in MeCN and CH_2_Cl_2_ were below the detection limit of our instruments (<10 ns), supporting that the emission of **NIphen** occurs from a ^1^LC state. However, TA spectroscopy revealed much longer lifetimes of the dark excited states of **NIphen** in MeCN and CH_2_Cl_2_ (8.40 and 11.04 µs, respectively). The fact that both lifetimes are fairly similar suggests the presence of a ^3^LC state of the ligand. This is further supported by theoretical calculations *via* TD-DFT which predicted that the lowest triplet state of **NIphen** is entirely located on the naphthalimide moiety and should therefore have no charge transfer character (Supplementary Figure S35). (Yarnell et al., 2019) Considering also the moderate quantum yield of **NIphen** (see above), a population of the lower lying ^3^LC state is evident.

**FIGURE 4:**
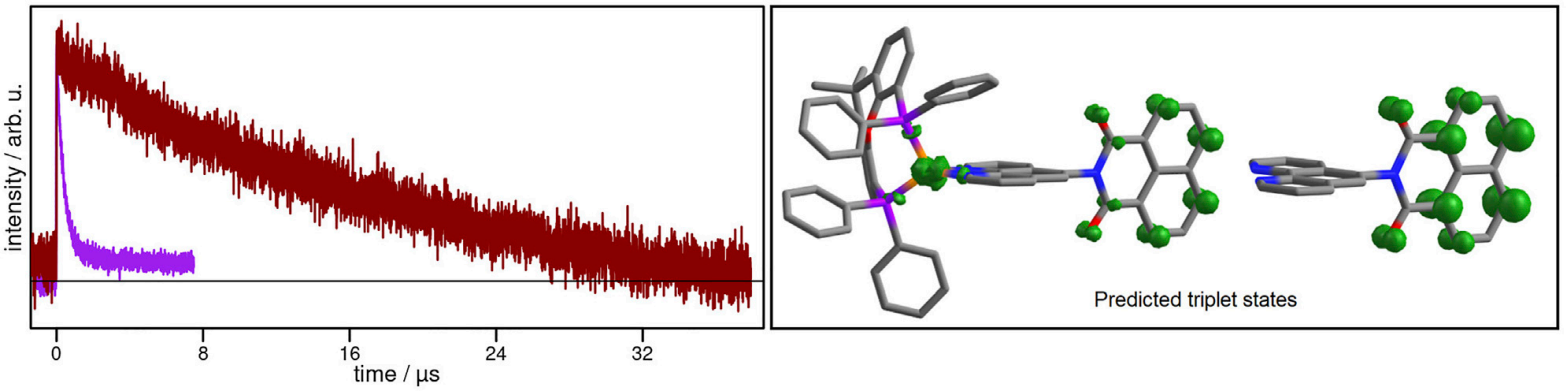
Left: Transient absorption spectra of **CuNIphen** at 480 nm in MeCN (dark red) and in CH_2_Cl_2_ (purple) solution under inert conditions excited at 355 nm. Right: Calculated spin density of the optimized lowest triplet state of **CuNIphen** simulated in CH_2_Cl_2_ (B3LYP-D3(BJ)/def2-tzvp, CPCM, isosurface value: 0.01). Please note that the identical calculation simulating MeCN (CPCM) yielded similar results (Supplementary Figure S35).

Next, transient signals could be obtained for the **CuNIphen** complex which is not emissive in deaerated MeCN. Kinetic analysis of these signals at 462, 468, 474 and 480 nm (Supplementary Figure S24) yielded an averaged exponential time constant of about 19.24 µs, consistent with the results obtained by Yarnell and co-workers (Yarnell et al., 2019). Therefore, in agreement with the results obtained for the **NIphen** ligand, the final occupied excited state in **CuNIphen** can be assigned to a dark ^3^LC state. Remarkably, the introduction of the Cu(I) center more than doubles the lifetime of the ^3^LC state in **CuNIphen** (19.24 µs) compared to **NIphen** (8.40 µs). Interestingly, the excited state lifetime decreases significantly from 19.24 µs in MeCN to 0.37 µs in CH_2_Cl_2_ (Figure 4), while the latter time is in the same range as for the structurally related **Cuphen** with 0.50 µs (Table 2). This process occurring in CH_2_Cl_2_ can therefore most probably be assigned to the depopulation of a ^3^MLCT state.

Similar to **CuNIphen** in MeCN, the long-lived non-emissive component of **Cubiipo** is attributed to the ^3^LC state of the **biipo** ligand with an excited state lifetime of 2.6 µs (Argüello Cordero et al., 2022). However, in CH_2_Cl_2_ solution **Cubiipo** still shows no emission and conversely reveals an increase of the time constant to 128.39 µs, which is in strong contrast to the here obtained results for **CuNIphen**.

### Singlet Oxygen Measurements

As known from our previous work, **Cubiipo** causes a weak ^1^O_2_ emission in aerated MeCN solution (Argüello Cordero et al., 2022). As evidenced by the TA measurements above, the lifetime of the long-lived component of **Cubiipo** increases dramatically from 2.6 µs in MeCN to 128.39 µs in CH_2_Cl_2_. Therefore, it is of great interest to evaluate the singlet oxygen (^1^O_2_) productivity of **Cubiipo** and **CuNIphen** in MeCN and also in CH_2_Cl_2_. To test the ^1^O_2_ productivity, the emission quantum yield of ^1^O_2_ can be observed at about 1276 nm (Schmid et al., 2022; Chettri et al., 2021). For this purpose, phenalenone (PN) is a universal reference compound which can be used in various solvents (Flors and Nonell, 2006; Silva et al., 2013; Trivella et al., 2014; Godard et al., 2020; Kaye et al., 2021; Payne et al., 2022). As expected, the much longer triplet state lifetime of **Cubiipo** in CH_2_Cl_2_ greatly enhances the generation of ^1^O_2_ with singlet oxygen quantum yields of 0.98 with respect to PN (QY_PN_(CH_2_Cl_2_) = 0.98).(Schmidt et al., 1994; Gallavardin et al., 2012; Epelde-Elezcano et al., 2016; Godard et al., 2020). For both **CuNIphen** and **Cuphen**, ^1^O_2_ emission quantum yields of 0.32 and 0.38 were obtained in aerated CH_2_Cl_2_, which are intriguingly similar (Supplementary Figure S22). These ^1^O_2_ quantum yields of **CuNIphen** and **Cuphen** are in very good agreement with the corresponding ^3^MLCT lifetimes of 0.37 and 0.50 µs in CH_2_Cl_2_ (Table 2). This strongly suggests that in the case of **CuNIphen** and **Cuphen** the ^1^O_2_ generation originates from the respective ^3^MLCT excited state. In addition, **CuNIphen** was also tested in MeCN, but no singlet oxygen emission was observed under these conditions. One possible reason for this could be that the energy of the excited state does not match with the energy required to convert ^3^O_2_ to ^1^O_2_ (DeRosa and Crutchley, 2002).

**FIGURE 5:**
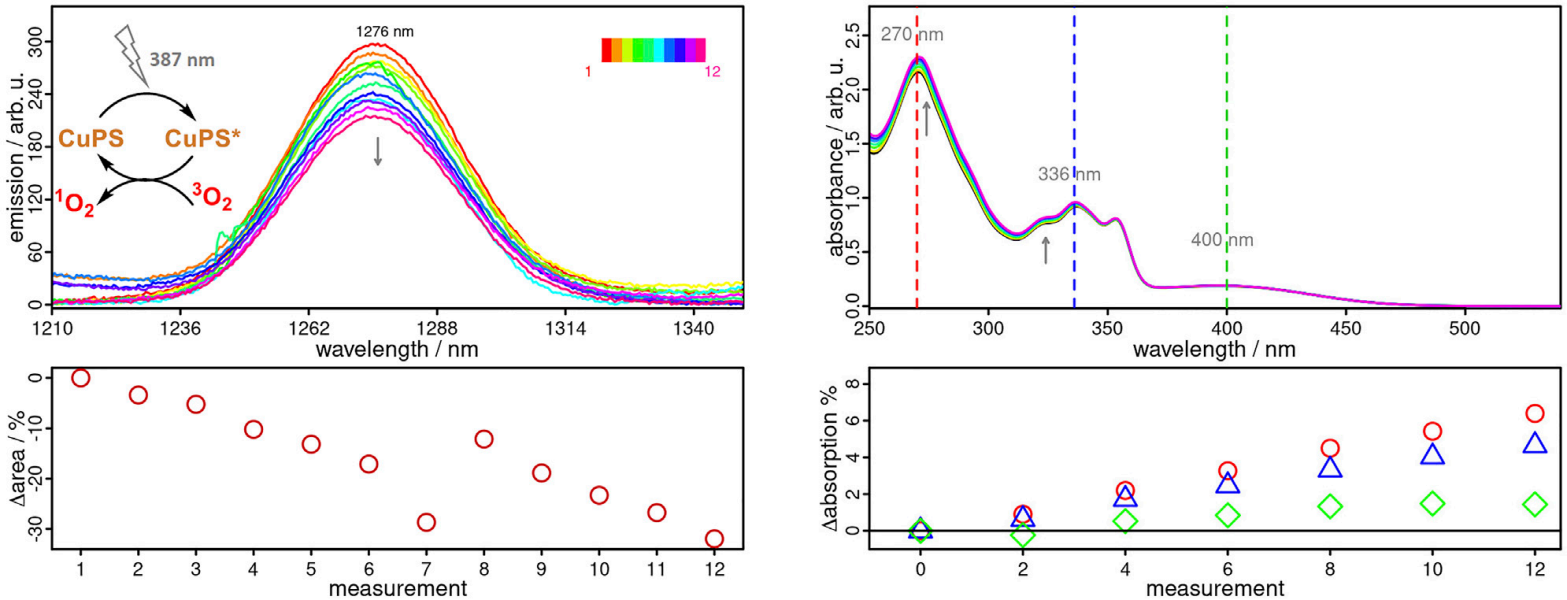
Left Top: Near-infrared emission spectra of **CuNIphen** in aerated deuterated dichloromethane solution (CD_2_Cl_2_) after excitation at 387 nm, showing the characteristic ^1^O_2_ emission at 1276 nm. The covered cuvette was shaken vigorously three times between each measurement. Left Bottom: Relative integral differences of the recorded ^1^O_2_ emission. Right Top: UV/vis absorption spectra of the same sample after every second emission measurement. Right Bottom: Relative differences in the absorbance of **CuNIphen** monitored at 270 (red), 336 (blue) and 400 nm (green), respectively.

Apart from a high activity, a high photostability is another crucial feature of a photosensitizer, not only for efficient ^1^O_2_ production, but also for continuous reuse without significant loss of activity (DeRosa and Crutchley, 2002). In this regard, **CuNIphen** exhibits an outstanding photostability over a period of 8.5 h in inert MeCN and of at least 1 h in aerated CH_2_Cl_2_ (Supplementary Figures S19, S20). These excellent properties motivated us to investigate the feasibility of continuous ^1^O_2_ production using **CuNIphen** in more detail. It is well known, that the use of deuterated solvents leads to much longer ^1^O_2_ lifetimes and more intense emissions compared to non-deuterated solvents (Bregnhøj et al., 2016), which we have also confirmed for our system (Supplementary Figure S22). Therefore, aerated CD_2_Cl_2_ was used to study the continuous ^1^O_2_ generation of **CuNIphen**. As indicated in Figure 5, **CuNIphen** produces ^1^O_2_ with almost constant activity over the course of 12 measurements. Moreover, only an insignificant decrease of the MLCT band (Δ = 1.4%, Figure 5) is observed, possibly due to photo bleaching by *in-situ* generated singlet oxygen or literature-known photo-induced ligand exchange processes (Kaeser et al., 2013; Fischer et al., 2014; Lennox et al., 2016). As described above, the high photostability of **CuNIphen** enables an efficient and continuous generation of ^1^O_2_. These properties of **CuNIphen** might open the door towards many different applications, such as in photoredoxcatalysis and photodynamic therapy (PDT).

## Conclusion

In this study, a systematic comparison between two different design strategies, namely between covalently linking and direct fusing of a naphthalimide unit, was performed. To this end, the heteroleptic Cu(I) photosensitizer **CuNIphen**, bearing a covalently linked naphthalimide substituent, and the unsubstituted reference complex **Cuphen** were prepared and fully characterized for the first time. The electrochemical, photophysical and catalytic properties were then evaluated in contrast to **Cubiipo**, which contains a directly fused naphthalimide unit. Analogous to **Cubiipo**, **CuNIphen** can be reversibly reduced twice, which means that the naphthalimide unit can accept a second electron. Nevertheless, the electronic communication between the phenanthroline moiety and the covalently attached naphtalimide unit is less pronounced in **CuNIphen** compared to fused π-system in **Cubiipo**. With respect to the mere **NIphen** ligand and the reference complex **Cuphen**, **CuNIphen** exhibits a much stronger absorption and increased attenuation coefficients over the whole spectrum.

The excited state features and associated kinetics were systematically investigated by a combination of emission and transient absorption spectroscopy in different solvents. In MeCN solution, **CuNIphen** exhibits a long-lived excited ^3^LC state with an excited state lifetime of 19.24 µs. In CH_2_Cl_2_, however, the lifetime drastically shortens to 0.37 µs, with the corresponding exited state most likely being ^3^MLCT in nature. In strong contrast to **CuNIphen**, the corresponding excited state lifetime of **Cubiipo** increases from 2.6 µs in MeCN to 128.39 µs in CH_2_Cl_2_, which causes significant changes in the catalytic behavior.

All three Cu(I) complexes were successfully applied in the light-driven formation of singlet oxygen (^1^O_2_) and the efficiency of ^1^O_2_ generation was directly evaluated by NIR emission spectroscopy. The resulting ^1^O_2_ quantum yields are consistent with the different excited state lifetimes from the TA measurements. More importantly, the exceptionally high photostability of **CuNIphen** enables a continuous ^1^O_2_ production. Over the course of 12 measurements, only a negligible decrease of the MLCT band (1.4%) of **CuNIphen** was detected. This renders **CuNIphen** as a promising candidate for further applications in the conversion of solar energy.

## Data Availability

The datasets presented in this study can be found in online repositories. CCDC 2165289 and CCDC 2165279 contain the supplementary crystallographic data for this paper. These data can be obtained free of charge by the Cambridge Crystallographic Data Centre *via* www.ccdc.cam.ac.uk/structures.

## References

[B1] Accorsi G. Listorti A. Yoosaf K. Armaroli N. (2009). 1,10-Phenanthrolines: Versatile Building Blocks for Luminescent Molecules, Materials and Metal Complexes. Chem. Soc. Rev. 38, 1690–1700. 10.1039/B806408N 19587962

[B2] Argüello Cordero M. A. Boden P. J. Rentschler M. Di Martino-Fumo P. Frey W. Yang Y. (2022). Comprehensive Picture of the Excited State Dynamics of Cu(I)- and Ru(II)-Based Photosensitizers with Long-Lived Triplet States. Inorg. Chem. 61, 214–226. 10.1021/acs.inorgchem.1c02771 34908410

[B3] Armaroli N. Balzani V. (2016). Solar Electricity and Solar Fuels: Status and Perspectives in the Context of the Energy Transition. Chem. Eur. J. 22, 32–57. 10.1002/chem.201503580 26584653

[B4] Armaroli N. (2001). Photoactive Mono- and Polynuclear Cu(i)-Phenanthrolines. A Viable Alternative to Ru(ii)-Polypyridines? Chem. Soc. Rev. 30, 113–124. 10.1039/B000703J

[B5] Au V. K.-M. (2021). Organic Light-Emitting Diodes Based on Luminescent Self-Assembled Materials of Copper(I). Energy Fuels 35, 18982–18999. 10.1021/acs.energyfuels.1c01956

[B6] Berardi S. Drouet S. Francàs L. Gimbert-Suriñach C. Guttentag M. Richmond C. (2014). Molecular Artificial Photosynthesis. Chem. Soc. Rev. 43, 7501–7519. 10.1039/C3CS60405E 24473472

[B7] Bregnhøj M. Westberg M. Jensen F. Ogilby P. R. (2016). Solvent-Dependent Singlet Oxygen Lifetimes: Temperature Effects Implicate Tunneling and Charge-Transfer Interactions. Phys. Chem. Chem. Phys. 18, 22946–22961. 10.1039/C6CP01635A 27484979

[B8] Büldt L. A. Wenger O. S. (2017). Chromium Complexes for Luminescence, Solar Cells, Photoredox Catalysis, Upconversion, and Phototriggered NO Release. Chem. Sci. 8, 7359–7367. 10.1039/C7SC03372A 29163886 PMC5672834

[B9] Call A. Cibian M. Yamamoto K. Nakazono T. Yamauchi K. Sakai K. (2019). Highly Efficient and Selective Photocatalytic CO2 Reduction to CO in Water by a Cobalt Porphyrin Molecular Catalyst. ACS Catal. 9, 4867–4874. 10.1021/acscatal.8b04975

[B10] Cao S. Low J. Yu J. Jaroniec M. (2015). Polymeric Photocatalysts Based on Graphitic Carbon Nitride. Adv. Mat. 27, 2150–2176. 10.1002/adma.201500033 25704586

[B11] Capaldo L. Ravelli D. (2020). The Dark Side of Photocatalysis: One Thousand Ways to Close the Cycle. Eur. J. Org. Chem. 2020, 2783–2806. 10.1002/ejoc.202000144

[B12] Castellano F. N. (2015). Altering Molecular Photophysics by Merging Organic and Inorganic Chromophores. Acc. Chem. Res. 48, 828–839. 10.1021/ar500385e 25646710

[B13] Chen N. Y. Xia L. M. Lennox A. J. J. Sun Y. Y. Chen H. Jin H. M. (2017). Structure-Activated Copper Photosensitisers for Photocatalytic Water Reduction. Chem. Eur. J. 23, 3631–3636. 10.1002/chem.201602598 27981644

[B14] Chen Y.-Z. Li W.-H. Li L. Wang L.-N. (2018). Progress in Organic Photocatalysts. Rare Mater. 37, 1–12. 10.1007/s12598-017-0953-2

[B15] Chettri A. Roque J. A. III Schneider K. R. A. Cole H. D. Cameron C. G. McFarland S. A. (2021). It Takes Three to Tango: The Length of the Oligothiophene Chain Determines the Nature of the Long-Lived Excited State and the Resulting Photocytotoxicity of a Ruthenium(II) Photodrug. ChemPhotoChem 5, 421–425. 10.1002/cptc.202000283 34337147 PMC8323708

[B16] Chi Y. Chou P.-T. (2010). Transition-Metal Phosphors with Cyclometalating Ligands: Fundamentals and Applications. Chem. Soc. Rev. 39, 638–655. 10.1039/B916237B 20111785

[B17] Colombo A. Dragonetti C. Roberto D. Fagnani F. (2021). Copper Complexes as Alternative Redox Mediators in Dye-Sensitized Solar Cells. Molecules 26, 194. 10.3390/molecules26010194 PMC7796243 33401723

[B18] DeRosa M. Crutchley R. (2002). Photosensitized Singlet Oxygen and its Applications. Coord. Chem. Rev. 233-234, 351–371. 10.1016/S0010-8545(02)00034-6

[B19] Detz R. J. Reek J. N. H. van der Zwaan B. C. C. (2018). The Future of Solar Fuels: When Could They Become Competitive? Energy Environ. Sci. 11, 1653–1669. 10.1039/C8EE00111A

[B20] Doettinger F. Yang Y. Schmid M.-A. Frey W. Karnahl M. Tschierlei S. (2021). Cross-Coupled Phenyl- and Alkynyl-Based Phenanthrolines and Their Effect on the Photophysical and Electrochemical Properties of Heteroleptic Cu(I) Photosensitizers. Inorg. Chem. 60, 5391–5401. 10.1021/acs.inorgchem.1c00416 33764043

[B21] Dong J. Zhang Y. Hussain M. I. Zhou W. Chen Y. Wang L.-N. (2022). g-C3N4: Properties, Pore Modifications, and Photocatalytic Applications. Nanomaterials 12, 121. 10.3390/nano12010121 PMC8746910 35010072

[B22] Dragonetti C. Magni M. Colombo A. Fagnani F. Roberto D. Melchiorre F. (2019). Towards Efficient Sustainable Full-Copper Dye-Sensitized Solar Cells. Dalton Trans. 48, 9703–9711. 10.1039/C9DT00790C 30969290

[B23] Dragonetti C. Magni M. Colombo A. Melchiorre F. Biagini P. Roberto D. (2018). Coupling of a Copper Dye with a Copper Electrolyte: A Fascinating Springboard for Sustainable Dye-Sensitized Solar Cells. ACS Appl. Energy Mat. 1, 751–756. 10.1021/acsaem.7b00196

[B24] Eckenhoff W. T. Eisenberg R. (2012). Molecular Systems for Light Driven Hydrogen Production. Dalton Trans. 41, 13004–13021. 10.1039/C2DT30823A 23014879

[B25] Epelde-Elezcano N. Martínez-Martínez V. Peña-Cabrera E. Gómez-Durán C. F. A. Arbeloa I. L. Lacombe S. (2016). Modulation of Singlet Oxygen Generation in Halogenated BODIPY Dyes by Substitution at Their Meso Position: Towards a Solvent-Independent Standard in the Vis Region. RSC Adv. 6, 41991–41998. 10.1039/C6RA05820E

[B26] Fischer S. Hollmann D. Tschierlei S. Karnahl M. Rockstroh N. Barsch E. (2014). Death and Rebirth: Photocatalytic Hydrogen Production by a Self-Organizing Copper-Iron System. ACS Catal. 4, 1845–1849. 10.1021/cs500387e

[B27] Flors C. Nonell S. (2006). Light and Singlet Oxygen in Plant Defense against Pathogens: Phototoxic Phenalenone Phytoalexins. Acc. Chem. Res. 39, 293–300. 10.1021/ar0402863 16700528

[B28] Forero Cortés P. A. Marx M. Trose M. Beller M. (2021). Heteroleptic Copper Complexes with Nitrogen and Phosphorus Ligands in Photocatalysis: Overview and Perspectives. Chem. Catal. 1, 298–338. 10.1016/j.checat.2021.05.005

[B29] Frischmann P. D. Mahata K. Würthner F. (2013). Powering the Future of Molecular Artificial Photosynthesis with Light-Harvesting Metallosupramolecular Dye Assemblies. Chem. Soc. Rev. 42, 1847–1870. 10.1039/C2CS35223K 22850767

[B30] Gallavardin T. Armagnat C. Maury O. Baldeck P. L. Lindgren M. Monnereau C. (2012). An Improved Singlet Oxygen Sensitizer with Two-Photon Absorption and Emission in the Biological Transparency Window as a Result of Ground State Symmetry-Breaking. Chem. Commun. 48, 1689–1691. 10.1039/C2CC15904J 22182988

[B31] Georgiev N. I. Dimitrova M. D. Todorova Y. D. Bojinov V. B. (2016). Synthesis, Chemosensing Properties and Logic Behaviour of a Novel Ratiometric 1,8-Naphthalimide Probe Based on ICT and PET. Dyes Pigments 131, 9–17. 10.1016/j.dyepig.2016.03.051

[B32] Gernert M. Balles-Wolf L. Kerner F. Müller U. Schmiedel A. Holzapfel M. (2020). Cyclic (Amino)(aryl)carbenes Enter the Field of Chromophore Ligands: Expanded π System Leads to Unusually Deep Red Emitting CuI Compounds. J. Am. Chem. Soc. 142, 8897–8909. 10.1021/jacs.0c02234 32302135

[B33] Giereth R. Obermeier M. Forschner L. Karnahl M. Schwalbe M. Tschierlei S. (2021). Exploring the Full Potential of Photocatalytic Carbon Dioxide Reduction Using a Dinuclear Re2Cl2 Complex Assisted by Various Photosensitizers. ChemPhotoChem 5, 644–653. 10.1002/cptc.202100034

[B34] Giereth R. Reim I. Frey W. Junge H. Tschierlei S. Karnahl M. (2019). Remarkably Long-Lived Excited States of Copper Photosensitizers Containing an Extended π-System Based on an Anthracene Moiety. Sustain. Energy Fuels 3, 692–700. 10.1039/c8se00521d

[B35] Godard J. Brégier F. Arnoux P. Myrzakhmetov B. Champavier Y. Frochot C. (2020). New Phenalenone Derivatives: Synthesis and Evaluation of Their Singlet Oxygen Quantum Yield. ACS Omega 5, 28264–28272. 10.1021/acsomega.0c04172 33163810 PMC7643266

[B36] Gür T. M. (2018). Review of Electrical Energy Storage Technologies, Materials and Systems: Challenges and Prospects for Large-Scale Grid Storage. Energy Environ. Sci. 11, 2696–2767. 10.1039/C8EE01419A

[B37] Hagfeldt A. Boschloo G. Sun L. Kloo L. Pettersson H. (2010). Dye-Sensitized Solar Cells. Chem. Rev. 110, 6595–6663. 10.1021/cr900356p 20831177

[B38] Hammarström L. (2016). Catalyst: Chemistry's Role in Providing Clean and Affordable Energy for All. Chem 1, 515–518. 10.1016/j.chempr.2016.09.002

[B39] Heberle M. Tschierlei S. Rockstroh N. Ringenberg M. Frey W. Junge H. (2017). Heteroleptic Copper Photosensitizers: Why an Extended π-System Does Not Automatically Lead to Enhanced Hydrogen Production. Chem. Eur. J. 23, 312–319. 10.1002/chem.201604005 27768809

[B40] Kaeser A. Mohankumar M. Mohanraj J. Monti F. Holler M. Cid J.-J. (2013). Heteroleptic Copper(I) Complexes Prepared from Phenanthroline and Bis-Phosphine Ligands. Inorg. Chem. 52, 12140–12151. 10.1021/ic4020042 24083360

[B41] Kaye E. G. Kailass K. Sadovski O. Beharry A. A. (2021). A Green-Absorbing, Red-Fluorescent Phenalenone-Based Photosensitizer as a Theranostic Agent for Photodynamic Therapy. ACS Med. Chem. Lett. 12, 1295–1301. 10.1021/acsmedchemlett.1c00284 34413959 PMC8366004

[B42] Keller S. Prescimone A. La Placa M.-G. Junquera-Hernández J. M. Bolink H. J. Constable E. C. (2020). The Shiny Side of Copper: Bringing Copper(i) Light-Emitting Electrochemical Cells Closer to Application. RSC Adv. 10, 22631–22644. 10.1039/D0RA03824E 35514545 PMC9054616

[B43] Kim J. Whang D. R. Park S. Y. (2017). Designing Highly Efficient Cu I Photosensitizers for Photocatalytic H 2 Evolution from Water. ChemSusChem 10, 1883–1886. 10.1002/cssc.201700389 28332772

[B44] Kuang S.-M. Cuttell D. G. McMillin D. R. Fanwick P. E. Walton R. A. (2002). Synthesis and Structural Characterization of Cu(I) and Ni(II) Complexes that Contain the Bis[2-(diphenylphosphino)phenyl]ether Ligand. Novel Emission Properties for the Cu(I) Species. Inorg. Chem. 41, 3313–3322. 10.1021/ic0201809 12055011

[B45] Lang X. Chen X. Zhao J. (2014). Heterogeneous Visible Light Photocatalysis for Selective Organic Transformations. Chem. Soc. Rev. 43, 473–486. 10.1039/C3CS60188A 24162830

[B46] Lazorski M. S. Castellano F. N. (2014). Advances in the Light Conversion Properties of Cu(I)-Based Photosensitizers. Polyhedron 82, 57–70. 10.1016/j.poly.2014.04.060

[B47] Leis W. Argüello Cordero M. A. Lochbrunner S. Schubert H. Berkefeld A. (2022). A Photoreactive Iron(II) Complex Luminophore. J. Am. Chem. Soc. 144, 1169–1173. 10.1021/jacs.1c13083 35025493

[B48] Lennox A. J. J. Fischer S. Jurrat M. Luo S.-P. Rockstroh N. Junge H. (2016). Copper-Based Photosensitisers in Water Reduction: A More Efficient *In Situ* Formed System and Improved Mechanistic Understanding. Chem. Eur. J. 22, 1233–1238. 10.1002/chem.201503812 26691442

[B49] Leoni E. Mohanraj J. Holler M. Mohankumar M. Nierengarten I. Monti F. (2018). Heteroleptic Copper(I) Complexes Prepared from Phenanthroline and Bis-Phosphine Ligands: Rationalization of the Photophysical and Electrochemical Properties. Inorg. Chem. 57, 15537–15549. 10.1021/acs.inorgchem.8b02879 30481016

[B50] Lewis N. S. (2016). Research Opportunities to Advance Solar Energy Utilization. Science 351, aad1920. 10.1126/science.aad1920 26798020

[B51] Luo S.-P. Mejía E. Friedrich A. Pazidis A. Junge H. Surkus A.-E. (2013). Photocatalytic Water Reduction with Copper-Based Photosensitizers: A Noble-Metal-Free System. Angew. Chem. Int. Ed. 52, 419–423. 10.1002/anie.201205915 23047871

[B52] McCullough B. J. Neyhouse B. J. Schrage B. R. Reed D. T. Osinski A. J. Ziegler C. J. (2018). Visible-Light-Driven Photosystems Using Heteroleptic Cu(I) Photosensitizers and Rh(III) Catalysts to Produce H2. Inorg. Chem. 57, 2865–2875. 10.1021/acs.inorgchem.7b03273 29446925

[B53] Mejía E. Luo S.-P. Karnahl M. Friedrich A. Tschierlei S. Surkus A.-E. (2013). A Noble-Metal-Free System for Photocatalytic Hydrogen Production from Water. Chem. Eur. J. 19, 15972–15978. 10.1002/chem.201302091 24123302

[B54] Nalzala Thomas M. R. Kanniyambatti Lourdusamy V. J. Dhandayuthapani A. A. Jayakumar V. (2021). Non-Metallic Organic Dyes as Photosensitizers for Dye-Sensitized Solar Cells: A Review. Environ. Sci. Pollut. Res. 28, 28911–28925. 10.1007/s11356-021-13751-7 33856633

[B55] Obermeier M. Beckmann F. Schaer R. S. Wenger O. S. Schwalbe M. (2021). Sensitized Photocatalytic CO2 Reduction with Earth Abundant 3d Metal Complexes Possessing Dipicolyl-Triazacyclononane Derivatives. Front. Chem. 9, 751716. 10.3389/fchem.2021.751716 34660540 PMC8514774

[B56] Paria S. Reiser O. (2014). Copper in Photocatalysis. ChemCatChem 6, 2477–2483. 10.1002/cctc.201402237

[B57] Paria S. Reiser O. (2018). Visible Light and Copper Complexes: A Promising Match in Photoredox Catalysis. Weinheim, Germany: Wiley-VCH Verlag GmbH & Co., 233–251. 10.1002/9783527674145.ch7

[B58] Payne D. T. Hynek J. Labuta J. Hill J. P. (2022). Nonionic Omnisoluble Photosensitizer Reference Material for the Estimation of Singlet Oxygen Quantum Yield. Phys. Chem. Chem. Phys. 24, 6146–6154. 10.1039/D1CP04651A 35225308

[B59] Penu R. Litescu S. C. Eremia S. A. V. Vasilescu I. Radu G.-L. Giardi M. T. (2015). Application of an Optimized Electrochemical Sensor for Monitoring Astaxanthin Antioxidant Properties against Lipoperoxidation. New J. Chem. 39, 6428–6436. 10.1039/C5NJ00457H

[B60] Prier C. K. Rankic D. A. MacMillan D. W. C. (2013). Visible Light Photoredox Catalysis with Transition Metal Complexes: Applications in Organic Synthesis. Chem. Rev. 113, 5322–5363. 10.1021/cr300503r 23509883 PMC4028850

[B61] Reichenauer F. Wang C. Förster C. Boden P. Ugur N. Báez-Cruz R. (2021). Strongly Red-Emissive Molecular Ruby [Cr(bpmp)2]3+ Surpasses [Ru(bpy)3]2+. J. Am. Chem. Soc. 143, 11843–11855. 10.1021/jacs.1c05971 34296865

[B62] Reiß B. Wagenknecht H.-A. (2019). Naphthalene Diimides with Improved Solubility for Visible Light Photoredox Catalysis. Beilstein J. Org. Chem. 15, 2043–2051. 10.3762/bjoc.15.201 31501672 PMC6720061

[B63] Rentschler M. Schmid M.-A. Frey W. Tschierlei S. Karnahl M. (2020). Multidentate Phenanthroline Ligands Containing Additional Donor Moieties and Their Resulting Cu(I) and Ru(II) Photosensitizers: A Comparative Study. Inorg. Chem. 59, 14762–14771. 10.1021/acs.inorgchem.9b03687 32212646

[B64] Schmid M.-A. Rentschler M. Frey W. Tschierlei S. Karnahl M. (2018). Imidazo-Phenanthroline Ligands as a Convenient Modular Platform for the Preparation of Heteroleptic Cu(I) Photosensitizers. Inorganics 6, 134. 10.3390/inorganics6040134

[B65] Schmid M. A. Brückmann J. Bösking J. Nauroozi D. Karnahl M. Rau S. (2022). Merging of a Perylene Moiety Enables a Ru II Photosensitizer with Long-Lived Excited States and the Efficient Production of Singlet Oxygen. Chem. Eur. J. 28, e202103609. 10.1002/chem.202103609 34767288 PMC9299699

[B66] Schmidt R. Tanielian C. Dunsbach R. Wolff C. (1994). Phenalenone, a Universal Reference Compound for the Determination of Quantum Yields of Singlet Oxygen O2(1Δg) Sensitization. J. Photochem. Photobiol. A Chem. 79, 11–17. 10.1016/1010-6030(93)03746-4

[B67] Schultz D. M. Yoon T. P. (2014). Solar Synthesis: Prospects in Visible Light Photocatalysis. Science 343, 1239176. 10.1126/science.1239176 24578578 PMC4547527

[B68] Schulz M. Hagmeyer N. Wehmeyer F. Lowe G. Rosenkranz M. Seidler B. (2020). Photoinduced Charge Accumulation and Prolonged Multielectron Storage for the Separation of Light and Dark Reaction. J. Am. Chem. Soc. 142, 15722–15728. 10.1021/jacs.0c03779 32830491

[B69] Silva E. F. F. Schaberle F. A. Monteiro C. J. P. Dąbrowski J. M. Arnaut L. G. (2013). The Challenging Combination of Intense Fluorescence and High Singlet Oxygen Quantum Yield in Photostable Chlorins - a Contribution to Theranostics. Photochem. Photobiol. Sci. 12, 1187–1192. 10.1039/C3PP25419D 23584281

[B70] Soulis K. Gourlaouen C. Daniel C. Quatela A. Odobel F. Blart E. (2018). New Luminescent Copper(I) Complexes with Extended π-Conjugation. Polyhedron 140, 42–50. 10.1016/j.poly.2017.11.026

[B71] Steinlechner C. Roesel A. F. Oberem E. Päpcke A. Rockstroh N. Gloaguen F. (2019). Selective Earth-Abundant System for CO2 Reduction: Comparing Photo- and Electrocatalytic Processes. ACS Catal. 9, 2091–2100. 10.1021/acscatal.8b03548

[B72] Stephen Nalley A. (2021). Annual Energy Outlook 2021.

[B73] Suraru S.-L. Würthner F. (2014). Strategies for the Synthesis of Functional Naphthalene Diimides. Angew. Chem. Int. Ed. 53, 7428–7448. 10.1002/anie.201309746 24961807

[B74] Szakács Z. Bojtár M. Hessz D. Rousseva S. Bitter I. Drahos L. (2019). Strong Ion Pair Charge Transfer Interaction of 1,8-Naphthalimide-Bipyridinium Conjugates with Basic Anions - towards the Development of a New Type of Turn-On Fluorescent Anion Sensors. New J. Chem. 43, 6666–6674. 10.1039/C9NJ00382G

[B75] Takeda H. Ohashi K. Sekine A. Ishitani O. (2016). Photocatalytic CO2 Reduction Using Cu(I) Photosensitizers with a Fe(II) Catalyst. J. Am. Chem. Soc. 138, 4354–4357. 10.1021/jacs.6b01970 27015323

[B76] Teegardin K. Day J. I. Chan J. Weaver J. (2016). Advances in Photocatalysis: A Microreview of Visible Light Mediated Ruthenium and Iridium Catalyzed Organic Transformations. Org. Process Res. Dev. 20, 1156–1163. 10.1021/acs.oprd.6b00101 27499607 PMC4972501

[B77] Treiling S. Wang C. Förster C. Reichenauer F. Kalmbach J. Boden P. (2019). Luminescence and Light-Driven Energy and Electron Transfer from an Exceptionally Long-Lived Excited State of a Non-Innocent Chromium(III) Complex. Angew. Chem. Int. Ed. 58, 18075–18085. 10.1002/anie.201909325 PMC6916301 31600421

[B78] Trivella A. S. Monadjemi S. Worrall D. R. Kirkpatrick I. Arzoumanian E. Richard C. (2014). Perinaphthenone Phototransformation in a Model of Leaf Epicuticular Waxes. J. Photochem. Photobiol. B Biol. 130, 93–101. 10.1016/j.jphotobiol.2013.10.009 24300996

[B79] Tsubomura T. Kimura K. Nishikawa M. Tsukuda T. (2015). Structures and Photophysical Properties of Copper(i) Complexes Bearing Diphenylphenanthroline and Bis(Diphenylphosphino)alkane: The Effect of Phenyl Groups on the Phenanthroline Ligand. Dalton Trans. 44, 7554–7562. 10.1039/C5DT00835B 25804312

[B80] Tyson D. S. Luman C. R. Zhou X. Castellano F. N. (2001). New Ru(II) Chromophores with Extended Excited-State Lifetimes. Inorg. Chem. 40, 4063–4071. 10.1021/ic010287g 11466069

[B81] Volz D. Zink D. M. Bocksrocker T. Friedrichs J. Nieger M. Baumann T. (2013). Molecular Construction Kit for Tuning Solubility, Stability and Luminescence Properties: Heteroleptic MePyrPHOS-Copper Iodide-Complexes and Their Application in Organic Light-Emitting Diodes. Chem. Mat. 25, 3414–3426. 10.1021/cm4010807

[B82] Wegeberg C. Wenger O. S. (2021). Luminescent First-Row Transition Metal Complexes. JACS Au 1, 1860–1876. 10.1021/jacsau.1c00353 34841405 PMC8611671

[B83] Wells K. A. Yarnell J. E. Sheykhi S. Palmer J. R. Yonemoto D. T. Joyce R. (2021). Accessing the Triplet Manifold of Naphthalene Benzimidazole-Phenanthroline in Rhenium(i) Bichromophores. Dalton Trans. 50, 13086–13095. 10.1039/D1DT02329B 34581368

[B84] Wenger O. S. (2019). Is Iron the New Ruthenium? Chem. Eur. J. 25, 6043–6052. 10.1002/chem.201806148 30615242

[B85] Würthner F. Saha-Möller C. R. Fimmel B. Ogi S. Leowanawat P. Schmidt D. (2016). Perylene Bisimide Dye Assemblies as Archetype Functional Supramolecular Materials. Chem. Rev. 116, 962–1052. 10.1021/acs.chemrev.5b00188 26270260

[B86] Xiao H. Chen M. Shi G. Wang L. Yin H. Mei C. (2010). A Novel Fluorescent Molecule Based on 1,8-Naphthalimide: Synthesis, Spectral Properties, and Application in Cell Imaging. Res. Chem. Intermed. 36, 1021–1026. 10.1007/s11164-010-0214-6

[B87] Yang Y. Brückmann J. Frey W. Rau S. Karnahl M. Tschierlei S. (2020). Electron Storage Capability and Singlet Oxygen Productivity of a RuII Photosensitizer Containing a Fused Naphthaloylenebenzene Moiety at the 1,10-Phenanthroline Ligand. Chem. Eur. J. 26, 17027–17034. 10.1002/chem.202001564 32519770 PMC7820985

[B88] Yarnell J. E. Deaton J. C. McCusker C. E. Castellano F. N. (2011). Bidirectional "Ping-Pong" Energy Transfer and 3000-Fold Lifetime Enhancement in a Re(I) Charge Transfer Complex. Inorg. Chem. 50, 7820–7830. 10.1021/ic200974h 21761837

[B89] Yarnell J. E. Wells K. A. Palmer J. R. Breaux J. M. Castellano F. N. (2019). Excited-State Triplet Equilibria in a Series of Re(I)-Naphthalimide Bichromophores. J. Phys. Chem. B 123, 7611–7627. 10.1021/acs.jpcb.9b05688 31405284

[B90] Yuan Y.-J. Yu Z.-T. Chen D.-Q. Zou Z.-G. (2017). Metal-Complex Chromophores for Solar Hydrogen Generation. Chem. Soc. Rev. 46, 603–631. 10.1039/C6CS00436A 27808300

[B91] Zhang J. Ma H. (2019). Synthesis, Characterization, and Crystal Structures of Imides Condensed with P-Phenylamino(Phenyl) Amine and Fluorescence Property. Materials 12, 1873. 10.3390/ma12111873 PMC6600954 31185634

[B92] Zhang Y. Heberle M. Wächtler M. Karnahl M. Dietzek B. (2016). Determination of Side Products in the Photocatalytic Generation of Hydrogen with Copper Photosensitizers by Resonance Raman Spectroelectrochemistry. RSC Adv. 6, 105801–105805. 10.1039/C6RA21469J

[B93] Zhang Y. Schulz M. Wächtler M. Karnahl M. Dietzek B. (2018a). Heteroleptic Diimine-Diphosphine Cu(I) Complexes as an Alternative towards Noble-Metal Based Photosensitizers: Design Strategies, Photophysical Properties and Perspective Applications. Coord. Chem. Rev. 356, 127–146. 10.1016/j.ccr.2017.10.016

[B94] Zhang Y. Traber P. Zedler L. Kupfer S. Gräfe S. Schulz M. (2018b). Cu(i) vs. Ru(ii) Photosensitizers: Elucidation of Electron Transfer Processes within a Series of Structurally Related Complexes Containing an Extended π-system. Phys. Chem. Chem. Phys. 20, 24843–24857. 10.1039/C8CP04595J 30230487

[B95] Zhang Y. Zedler L. Karnahl M. Dietzek B. (2019). Excited-State Dynamics of Heteroleptic Copper(i) Photosensitizers and Their Electrochemically Reduced Forms Containing a Dipyridophenazine Moiety - A Spectroelectrochemical Transient Absorption Study. Phys. Chem. Chem. Phys. 21, 10716–10725. 10.1039/C9CP00412B 31086897

