# Supplementary material for: How the Way a Naphthalimide Unit is Implemented Affects the Photophysical and -catalytic Properties of Cu(I) Photosensitizers
Source: Front Chem. 2022 Jun 17;10:936863. doi: 10.3389/fchem.2022.936863 (PMC9247301; doi:10.3389/fchem.2022.936863)
Supplement: Supplementary file 1 [file DataSheet1.pdf]

Supplementary Material

Supporting Information - Table of Contents

1	Experimental Details	page	S2
2	Synthetic Details	page	S6
3	Structural Characterization of **CuNIphen** and **Cuphen**	page	S9
	3.1 NMR Spectra	page	S9
	3.2 MS Spectra	page	S14
	3.3 Crystallographic Data	page	S16
4	Cyclic Voltammograms	page	S18
5	Experimental UV/vis Absorption and Emission Spectra	page	S20
6	Transient Absorption Spectra	page	S23
7	Calculated Ground State Structures	page	S25
8	Calculated UV/vis Spectra, Differential Density Plots and Spin Densities	page	S26

1 Experimental Details

NMR spectroscopy. Nuclear magnetic resonance (NMR) spectra were measured by the analytical service of the Institute of Inorganic and Analytical Chemistry at the Technische Universität Braunschweig at 298 K with a Bruker Avance IIIHD 500 spectrometer operating at frequencies of 500 MHz (^{1}H), 126 MHz (^{13}C) and 203 MHz (^{31}P). The spectra were then processed using the TopSpin software (version 4.1.1). All spectra are referenced against the deuterated solvent as internal standard. Coupling constants *J* are represented as absolute values in Hz. Characterization of the NMR signal splittings is denoted using the following abbreviations: *s* = singlet, *d* = doublet, *t* = triplet, *dd* = doublet of doublets, *td* =triplet of doublets and *m* = multiplet. Quintet splitting is described as *quintet*.

Mass spectrometry. Mass spectrometric (MS) measurements were performed by the analytical service of the Institute of Organic Chemistry at the Technische Universität Braunschweig. High resolution mass spectra were measured using electrospray ionization (ESI) on an LTQ-Orbitrap Velos orbitrap mass analyser from ThermoFisher Scientific. Samples were dissolved in methanol spiked with 0.1 mg/mL tetradecyltrimethylammonium bromide. MS values are given as *m/z*.

X-ray analysis. Data acquisition for **CuNIphen**: A suitable crystal was fixed on a Teflon loop mounted on the goniometer head of a Bruker Kappa APEXII dual source diffractometer. X-ray intensities were generated by a Cu-Kα Incoatec Microfocus source (wavelength λ = 1.54178 Å) and detected on a APEXII CCD-detector. Data collection, cell refinement and data reduction were established by the Bruker APEXII Software Suite. The structure of **CuNIphen** was solved by Direct Methods and refined with the full-matrix least squares method using the SHELXL-97 program package.

Data acquisition for **Cuphen**: A single crystal of suitable quality for X-ray crystallography was mounted on a Hampton loop and placed in the cold (110 K) nitrogen gas stream on the diffractometer. The intensity data were collected on an Oxford Diffraction Xcalibur EOS instrument using graphite monochromated MoK_α radiation. The reflections were indexed, integrated and absorption corrections were applied as implemented in the CrysAlisPro software package.[1] The structures were solved employing the program SHELXT and refined anisotropically for all non-hydrogen atoms by full-matrix least squares on all F^2 using SHELXL software.[2] During refinement and analysis of the crystallographic data the programs Mercury, PLATON, and OLEX^2 were used[3], as well as SHELXL-97 program package.

CCDC 2165289 (**CuNIphen**) and CCDC 2165279 (**Cuphen**) contain the supplementary crystallographic data for this paper. These data can be obtained free of charge by the Cambridge Crystallographic Data Centre via www.ccdc.cam.ac.uk/data_request/cif or by emailing data_request@ccdc.cam.ac.uk or by contacting The Cambridge Crystallographic Data Centre, 12 Union Road, Cambridge CB2 1EZ, UK

[1] CrysAlisPro, Rigaku Oxford Diffraction, 2018.

[2] a) G. M. Sheldrick, *Acta Cryst.* **2015**, *A71*, 3–8; b) G. M. Sheldrick, *Acta Cryst.* **2015**, *C71*, 3–8

[3] a) C. F. Macrae, I. Sovago, S. J. Cottrell, P. T. A. Galek, P. McCabe, E. Pidcock, M. Platings, G. P. Shields, J. S. Stevens, M. Towler, P. A. Wood, *J. Appl. Cryst.* **2020**, *53*, 226–235; b) A. L. Spek, *Acta Cryst.* **2009**, *D65*, 148–155; c) O. V. Dolomanov, L. J. Bourhis, R. J. Gildea, J. A. K. Howard, H. Puschmann, *J. Appl Crystallogr.* **2009**, *42*, 339–341.

Cyclic voltammetry. Cyclic voltammograms were measured in acetonitrile solution using 0.1 M Bu_4NPF_6 as the supporting electrolyte. An Autolab potentiostat PGSTAT204 from Metrohm was used with a three-electrode configuration. The working electrode was a glassy carbon disc with a 3 mm diameter stick and the counter electrode was a Pt wire. The reference electrode was a non-aqueous Ag/Ag^+ electrode (0.01 M $AgNO_3$ in acetonitrile) with the ferrocene/ferricenium (Fc/Fc^+) couple as external reference, which was added to the solution after each measurement. All potentials are reported versus the Fc/Fc^+ couple. All scan rates are 0.1 V/s unless otherwise noted.

Steady-state absorption and emission spectroscopy in solution. Steady-state UV/vis absorption spectra were recorded with a JASCO V-670 und JASCO V-770 spectrophotometers and emission spectra were measured with a Horiba Jobin-Yvon FluoroMax Plus-C automated benchtop fluorescence spectrometer. For measurements under air in acetonitrile, ROTISOLV®, UV/IR grade solvents purchased from Carl Roth, and dichloromethane, HPLC grade solvents from Fischer Chemical were used. The respective emission quantum yield of **NIphen** was calculated and referenced against the literature reported value for anthracene in deaerated ethanol as the standard of 0.28.[4]

Photostability tests. UV/vis absorption spectra were acquired with an Avantes AvaSpec-ULS2048CL spectrophotometer. A 150 W xenon lamp (LOT-QuantumDesign GmbH, LSE140/160.25C) was used as light source and 0.5 OD filter was introduced for the measurements. The measurements were carried out under oxygen free conditions by using degassed acetonitrile. Under aerated conditions by using dichloromethane a 360 nm long pass filter was implemented. The samples were prepared using the same method as for the absorption and emission measurements applying sealed quartz glass cuvettes with a pathlength of 10 mm.

Nanosecond transient absorption spectroscopy. Excitation pulses were generated using a Q-switched pulsed Nd:YAG laser (Q-smart 450mJ, Quantel laser) with an output centered at 355 nm (approx. 6 ns pulse duration, repetition rate of 10 Hz). The pulses were passed through a laser line filter (CWL = 355 ± 2 nm, FWHM = 10 ± 2 nm) to ensure that the samples were only excited by 355 nm light. The power of the pump beam was about 3 mJ per pulse at the sample. The stability of the sample was verified by means of UV/Vis spectra before and after each measurement. The spectrometer used was a LP980-K spectrometer from Edinburgh Instruments, where the pump and probe beams spatially overlapped at the sample position in a perpendicular beam setup. The probe lamp was operated in flash mode (150 W ozone-free xenon arc lamp, 30 A). After passing the sample the probe light was recorded using a photo multiplier tube (Hamamatsu R928P). A standard fused silica cuvette with a layer thickness of 10 mm and a sample OD of approximately 0.3 at the pump wavelength was used in this setup. The compounds, **NIphen**, **CuNIPhen, Cuphen** and **Cubiipo**, were dissolved in acetonitrile (Carl Roth, ROTISOLV®, UV/IR grade solvents) and in dichloromethane (Fischer Chemical, HPLC grade solvents) under inert conditions and under aerated conditions.

Emission lifetime. Measurements were performed using a Q-switched pulsed Nd:YAG laser (Q-smart 450 mJ, Quantel laser) with pulse durations of approx. 6 ns at a repetition rate of 10 Hz. As excitation pulses the Nd:YAG output centered at 355 nm were used. Afterwards, the excitation light additionally passed a laser line filter (CWL = 355 ± 2 nm, FWHM = 10 ± 2 nm) to exclude excitation by light with wavelengths of lower order which can be present due to harmonic generation. The power of the pump beam was about 1.0 mJ per pulse at the sample and a sample OD of approximately 0.1 at the pump wavelength was used. The emission lifetime of the samples was measured at their respective emission

---

[4] K. Suzuki, A. Kobayashi, et al. *Phys. Chem. Chem. Phys.*, **2009**, 11, 9850–9860.

maxima. The emitted light was recorded using a photo multiplier tube (Hamamatsu R928P) of the LP980 spectrometer (Edinburgh Instruments).

Singlet oxygen measurement. For evaluation of the singlet oxygen quantum yield, the phosphorescence of $^1O_2$ at approximately 1276 nm was detected with a Horiba Jobin-Yvon FluoroMax Plus-C automated benchtop spectrofluorometer equipped with a 150 W Xe arc excitation lamp, a R13456 photomultiplier tube detector (190-930 nm), a liquid-nitrogen cooled DSS-IGA020L InGaAs photodiode detector (800- 1550 nm) and Czerny-Turner monochromators with NIR grating blazed at 1000 nm. Absorption spectroscopy was measured with a JASCO Spectrometer V-770 and was done before and after each singlet oxygen measurement. Thus, stability of the respective complex on the timescale of the measurements was ensured. For each sample, emission spectra were recorded upon excitation at 387 nm. As a reference the whole procedure was repeated for the known standard phenalenone. The detected singlet oxygen emissions were corrected to 0 intensity at 1350 nm. After baseline correction for each measurement, the area below the signal was integrated. The respective singlet oxygen quantum yield $\varphi(^1O_2)$ was calculated and referenced against the literature reported value for phenalenone of 0.98±0.02.[5] The following equation was used:

$$\Phi_c = \Phi_R \left(\frac{A_R}{A_C}\right)\left(\frac{I_C}{I_R}\right)$$

In this equation, Φ_x is the quantum yield and A_x the absorbance of the respective substance (reference (R) phenalenone or compound (C)) and I_x the integral of the singlet oxygen emission. This was done for all three different optical densities and the final quantum yield was calculated as the average.[5,6]

The continuous $^1O_2$ production measurement was carried out in a 10 mm quartz cuvette with only half-filled deuterated dichloromethane solution making sure that there is enough air/O_2 inside. Between every record of the characteristic emission of $^1O_2$ the covered cuvette was vigorously shaken three times and the absorption spectra of the sample were obtained to ensure its photostability.

DFT calculations. Quantum chemical calculations at the density functional theory (DFT) level were performed utilizing the ORCA program package (Version 5.0.0).[7] Geometry optimizations of the electronic ground state were conducted using the BP86[8] exchange-correlation functional for preoptimization. The B3LYP[9] hybrid functional was used for final optimization and for TD-DFT calculations. Dispersion effects were accounted for using the D3 correction by S. Grimme including the Becke-Johnson (BJ) damping.[10] The Karlsruhe's valence triple-zeta polarization functions basis sets (def2-TZVP) were applied.[11] Solvation effects were treated with the conductor-like polarizable continuum model, CPCM.[12] Optimized geometries were verified as minima on the potential energy

---

5 a) N. Epelde-Elezcano, V. Martınez-Martınez. et al. *RSC Adv.*, **2016**, 6, 41991-41998; b) T. Gallavardin, C. Armagnat, et al. *Chem. Commun*., **2012**, 48, 1689–1691.

6 M.-A. Schmid, J. Brückmann, et al. *Chem. Eur*. J. **2022**, 28, e202103609.

7 F. Neese, F. Wennmohs, U. Becker, C. Riplinger, *J. Chem. Phys.* **2020** *152*, *224108*.

8 K. Eichkorn, O. Treutler, H. Öhm, M. Häser and R. Ahlrichs, *Chem. Phys. Lett.* **1995**, *242*, 652-660.

9 O. Treutler, R. Ahlrichs, *J. Chem. Phys*. **1995**, *102*, 346-354.

10 a) S. Grimme, J. Antony, S. Ehrlich, H. Krieg, *J. Chem. Phys.* **2010**, *132*, 154104. b) S. Grimme, S. Ehrlich, L. Goerigk, *J. Comput. Chem.* **2011**, *32*, 1456.

11 F. Weigend, R. Ahlrichs, *Phys. Chem. Chem. Phys.* **2005**, *7*, 3297-3305.

12 V. Barone, M. Cossi, *J. Phys. Chem. A*, **1998**, *102*, 1995.

surface by frequency calculations (analytical, B3LYP-D3(BJ)/def2-TZVP, CPCM). Visualizations of the B3LYP molecular orbitals and of the electron difference density plots were evoked using the Chemcraft software package (Version 1.8).[13]

[13] chemcraftprog.com

2 Synthetic Details

Reagents and Chemicals. All chemicals were purchased from commercial suppliers (*i.e.* Sigma-Aldrich, Carl-Roth and ABCR) and used as received. Acetonitrile (MeCN) and dichloromethane (CH_2Cl_2) were distilled over calcium hydride under argon atmosphere and stored under argon.

NIphen (2-(1,10-phenanthrolin-5-yl)-1H-benzo[de]isoquinoline-1,3(2H)-dione)

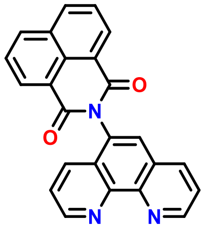

5-Naphthal-1,8-imido-1,10-phenanthroline
NIphen

The synthesis of 2-(1,10-phenanthrolin-5-yl)-1H-benzo[de]isoquinoline-1,3(2H)-dione (**NIphen**) was conducted like already described by Yarnell *et al*..[14] Analytical data (^{1}H NMR and HR-MS) were in accordance with the data described in the literature.

Synthesis of the complexes Cuphen and CuNIphen

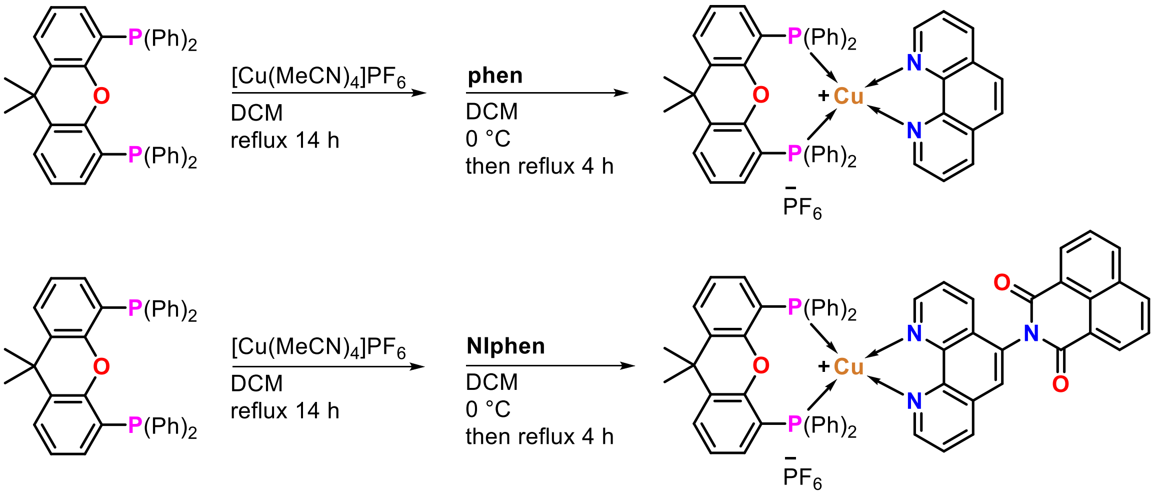

The complexes were synthesized following a literature known protocol[15]:

Into a schlenk-tube equipped with a magnetic stir bar were added tetrakis(acetonitrile)copper(I) hexafluorophosphate (74.5 mg, 0.2 mmol, 1 eq.) and (9,9-dimethyl-9*H*-xanthene-4,5-diyl)bis (diphenylphosphane) (115.7 mg, 0.2 mmol, 1 eq.). The vessel was attached to a reflux cooler and the

[14] J. Yarnell, K. Wells, J. Palmer, J. Breaux, F. Castellano. *J. Phys. Chem. B* **2019**, *123*, 7611−7627.

[15] a) M. Heberle and S. Tschierlei *et al.*, *Chem. Eur. J.* **2017**, *23*, 312-319. b) M. Rentschler, M.-A. Schmid, W. Frey. S. Tschierlei, M. Karnahl, *Inorg. Chem.* **2020**, *59*, 14762-14771.

whole apparatus was put under vacuum and refilled with argon three times. 20 ml of dry and degassed dichloromethane were added and the solution was refluxed for 14 hours.

The solution was then cooled to 0 °C and a solution of the respective phenanthroline-based ligand (36.0 mg **phen** or 80.7 mg **NIphen**, 0.2 mmol, 1 eq.) in 20 ml of dry and degassed dichloromethane was added drop wise very slowly (for **Cuphen** using a syringe pump at ca. 13 ml/h). After 30 min of stirring at 0°C the bright yellow solution was refluxed for four hours and then cooled to room temperature.

The complex was slowly precipitated by carefully adding *n*-hexane until the point where precipitation initiated and then stored at -20 °C overnight. The bright yellow crystalline precipitate was filtered off and washed with *n*-hexane and cold diethyl ether. **CuNIphen** was again dissolved in dichloromathane and reprecipitated with *n*-hexane twice to increase its purity. The solids were finally dried *in vacuo*.

CuNIphen: [(xant)Cu(**NIphen**)]PF_6: Yield: 92 mg (38 %)

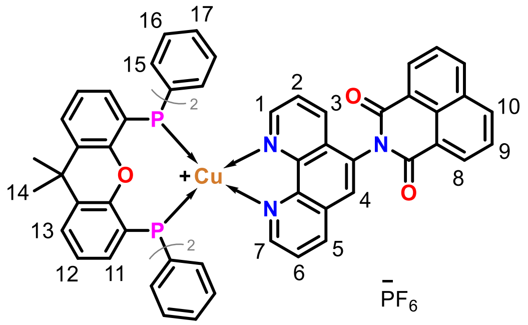

[(xant)Cu(**NIphen**)]PF_6
CuNIphen

$C_{63}H_{45}CuF_6N_3O_3P_3$ (M = 1162.52 g/mol). **^{1}H NMR** (CD_3CN, 500 MHz): δ = 8.65 (*m*, 3H, Ar-*H*, **7+8**); 8.57 (*d*, *J* = 8.0 Hz, 1H, Ar-*H*, **5**); 8.50 (*m*, 3H, Ar-*H*, **10+1**); 8.43 (*d*, *J* = 8.3 Hz, 1H, Ar-*H*, **3**); 8.18 (*s*, 1H, Ar-*H*, **4**); 7.93 (*t*, *J* = 7.8 Hz, 2H, Ar-*H*, **9**); 7.80 (*dd*, *J* = 7.8 Hz, *J* = 1.5 Hz, 2H, Ar-*H*, **13**); 7.73 (*dd*, *J* = 8.1 Hz, *J* = 4.8 Hz, 1H, Ar-*H*, **6**) ; 7.58 (*dd*, *J* = 8.1 Hz, *J* = 4.8 Hz, 1H, Ar-*H*, **2**); 7.30 (*t*, *J* = 7.5 Hz, 2H, Ar-*H*, **17**); 7.27 (*t*, *J* = 7.5 Hz, 2H, Ar-*H*, **17**); 7.22 (*t*, *J* = 7.7 Hz, 2H, Ar-*H*, **12**); 7.14 (*t*, *J* = 7.0 Hz, 4H, Ar-*H*, **16**); 7.11 (*t*, *J* = 7.0 Hz, 4H, Ar-*H*, **16**); 7.01 (*m*, 4H, Ar-*H*, **15**); 6.90 (*m*, 4H, Ar-*H*, **15**); 6.62 (*m*, 2H, Ar-*H*, **11**); 1.78 (*s*, 3H, CH_3, **14**); 1.74 (*s*, 3H, CH_3, **14**). **^{13}C NMR** (CD_3CN, 500 MHz): 165.49, 155.93 (*t*, *J* = 6.3 Hz), 151.28, 150.77, 145.02, 144.20, 139.10, 136.19, 135.32, 134.40, 133.74 (*m*), 133.08, 132.47, 132.45, 132.33, 132.19, 132.15, 131.19, 131.06, 130.13, 129.83, 129.80, 129.25, 128.95, 128.90, 128.41, 126.52, 126.37, 126.17, 123.84, 120.43 (*t*, *J* = 13.9 Hz), 37.09, 28.96, 28.20. **DEPT135** (CD_3CN, 500 MHz): 151.14, 150.62, 138.95, 136.04, 134.25, 133.59 (*m*), 132.30, 132.00, 130.98 (d, *J* = 18.7 Hz), 129.67 (*m*), 129.10, 128.75, 128.26, 126.31 (d, *J* = 18.0 Hz), 126.02 (*t*, *J* = 2.3 Hz), 28.81, 28.05. **^{31}P NMR** (CD_3CN, 500 MHz): -12.27 (*s*, Ar-*P*), -144.63 (*quintet*, *J* = 701 Hz, *P*F_6). **HRMS** (ESI) *m/z*: calcd. for $[C_{63}H_{45}CuN_3O_3P_2]^+$: 1016.2227, found: 1016.2208. **EA** (calc./found): C 65.09/65.13, H 3.90/3.94, N 3.61/3.59

Cuphen: [(xant)Cu(**phen**)]PF_6: Yield: 194 mg (80 %)

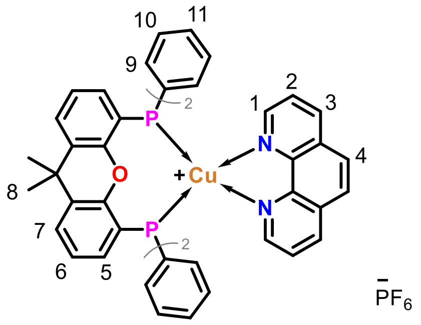

$C_{51}H_{40}CuF_6N_2OP_3$ (M = 967.35 g/mol). **^{1}H NMR** (CD_3CN, 500 MHz): 8.55 (*dd*, *J* = 8.2 Hz, *J* = 1.4 Hz, 2H, Ar-*H*, **3**), 8.52 (*d*, *J* = 4.2 Hz, 2H, Ar-*H*, **1**), 8.07 (*s*, 2H, Ar-*H*, **4**), 7.80 (*dd*, *J* = 7.9 Hz, *J* = 1.2 Hz, 2H, Ar-*H*, **7**), 7.68 (*dd*, *J* = 8.3 Hz, *J* = 4.8 Hz, 2H, Ar-*H*, **2**), 7.23 (*t*, *J* = 7.4 Hz, 4H, Ar-*H*, **11**), 7.21 (*t*, *J* = 7.8 Hz, 2H, Ar-*H*, **6**), 7.06 (*t*, *J* = 7.6 Hz, 8H, Ar-*H*, **10**), 6.92 (*q*, *J* = 6.0 Hz, 8H, Ar-*H*, **9**), 6.59 (*m*, 2H, **5**), 1.76 (*s*, 6H, C*H*$_3$, **8**). **^{13}C NMR** (CD_3CN, 500 MHz): 155.94 (*t*, *J* = 6.2 Hz), 150.41, 144.36, 138.77, 135.34, 133.72 (*t*, *J* = 8.1 Hz), 132.50 (*t*, *J* = 17.2 Hz), 132.11, 131.03, 130.91, 129.72 (*t*, *J* = 4.7 Hz), 128.85, 128.28, 126.12, 120.53 (*t*, *J* = 13.8 Hz), 37.10, 28.55. **^{31}P NMR** (CD_3CN, 500 MHz): -12.68 (*s*, Ar-*P*), -144.65 (*quintet*, *J* = 701 Hz, *P*F_6). **HRMS** (ESI) *m/z*: calcd. for $[C_{51}H_{40}CuN_2OP_2]^+$: 821.1906, found: 821.19159. **EA** (calc./found): C 63.32/62.44, H 4.17/4.31, N 2.90/2.88

3 Structural Characterization of CuNIphen and Cuphen

3.1 NMR Spectra

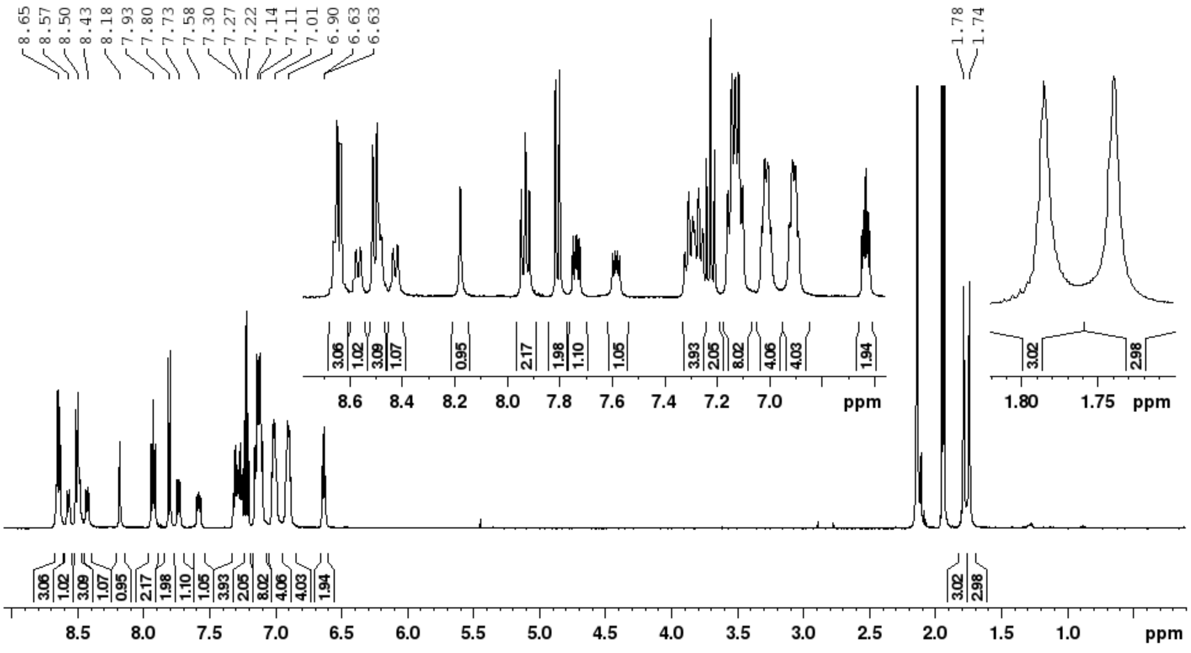

Supplementary Figure 1. ^{1}H NMR spectrum of **CuNIphen** (CD_3CN, 500 MHz).

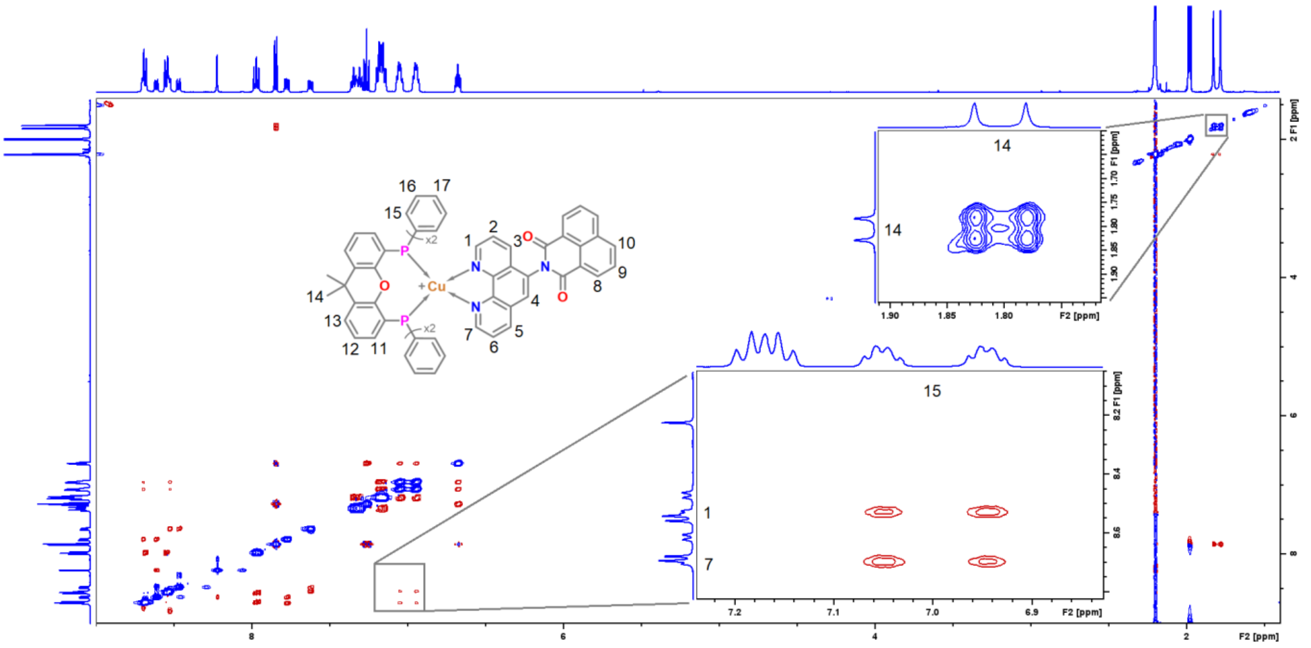

Supplementary Figure 2. NOESY-spectrum of **CuNIphen** (CD_3CN, 500 MHz). The upper inset magnifies the mutual interaction of the two methyl group's protons (14) inside the xantphos ligand backbone. The lower inset shows the interaction between the phenyl group´s *o*-protons (15) and the protons at 2- and 9-position (1 and 7) of the **NIphen** ligand.

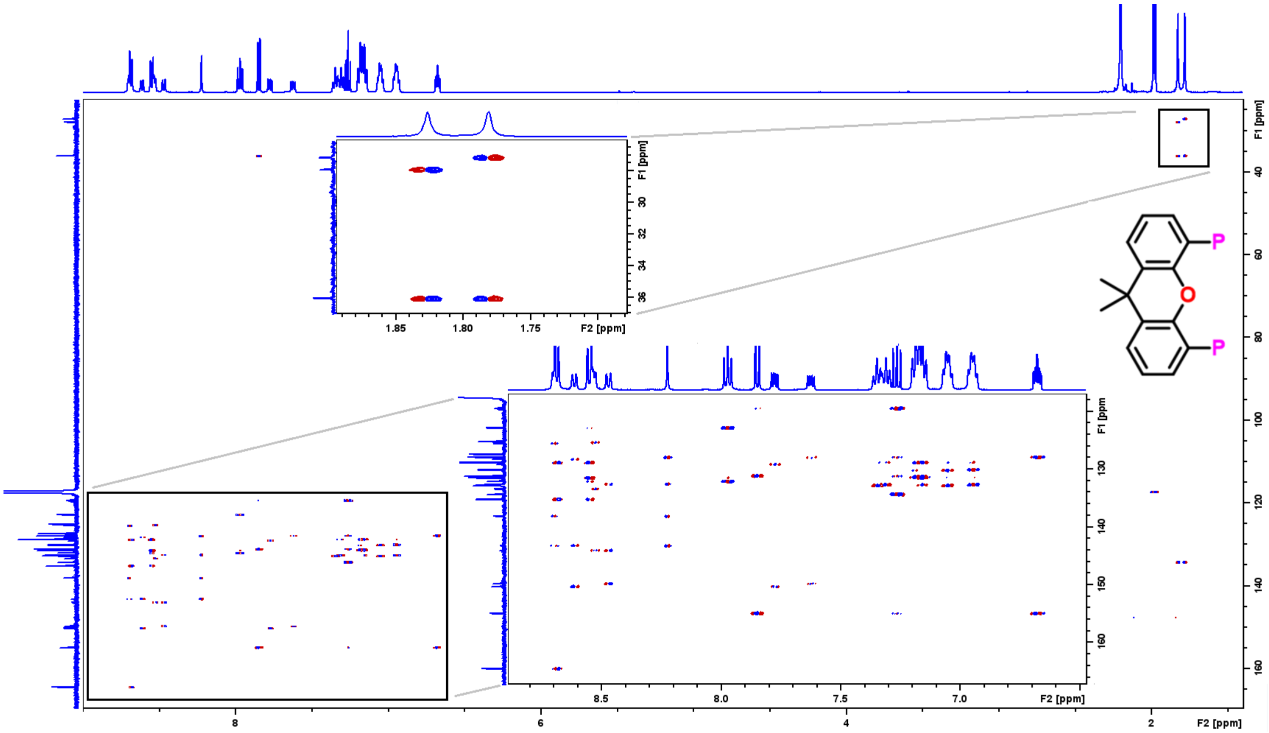

Supplementary Figure 3. HMBC-spectrum of **CuNIphen** (CD_3CN, 500 MHz). The upper inset shows that both groups of methyl protons at 1.78 ppm and 1.74 ppm (14) correspond to one carbon signal each (28.60 ppm and 28.20 ppm). There is also correlation over multiple bonds between the proton signals and the carbon atom connecting both methyl groups (*compare also structure fragment, right*).

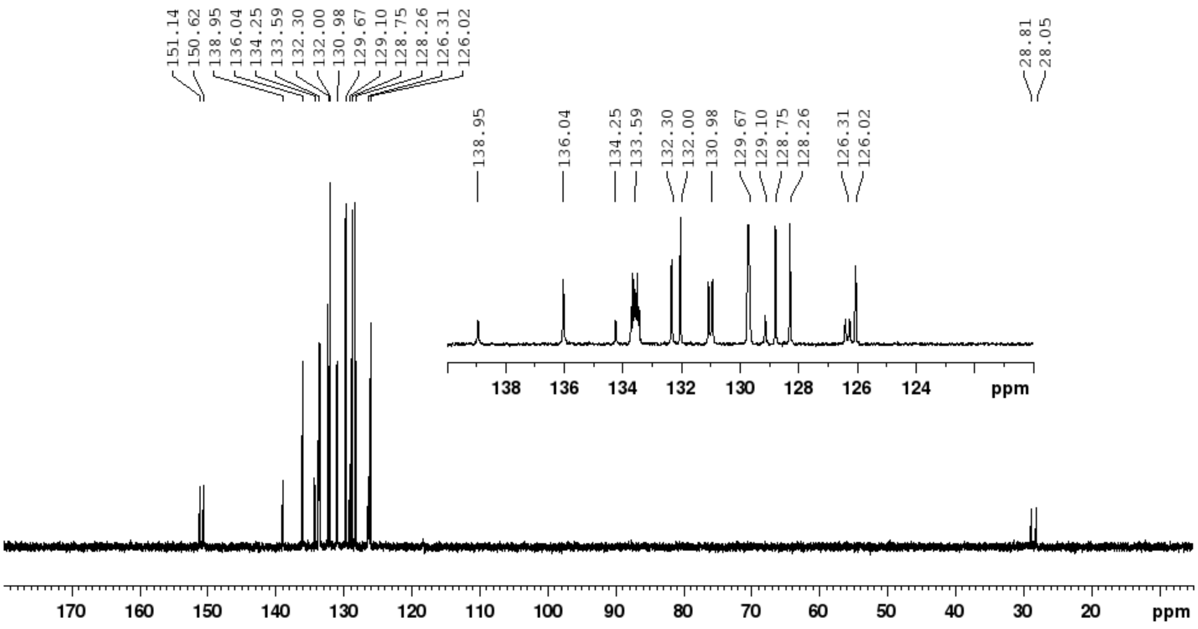

Supplementary Figure 4. DEPT135-spectrum of **CuNIphen** (CD_3CN, 500 MHz).

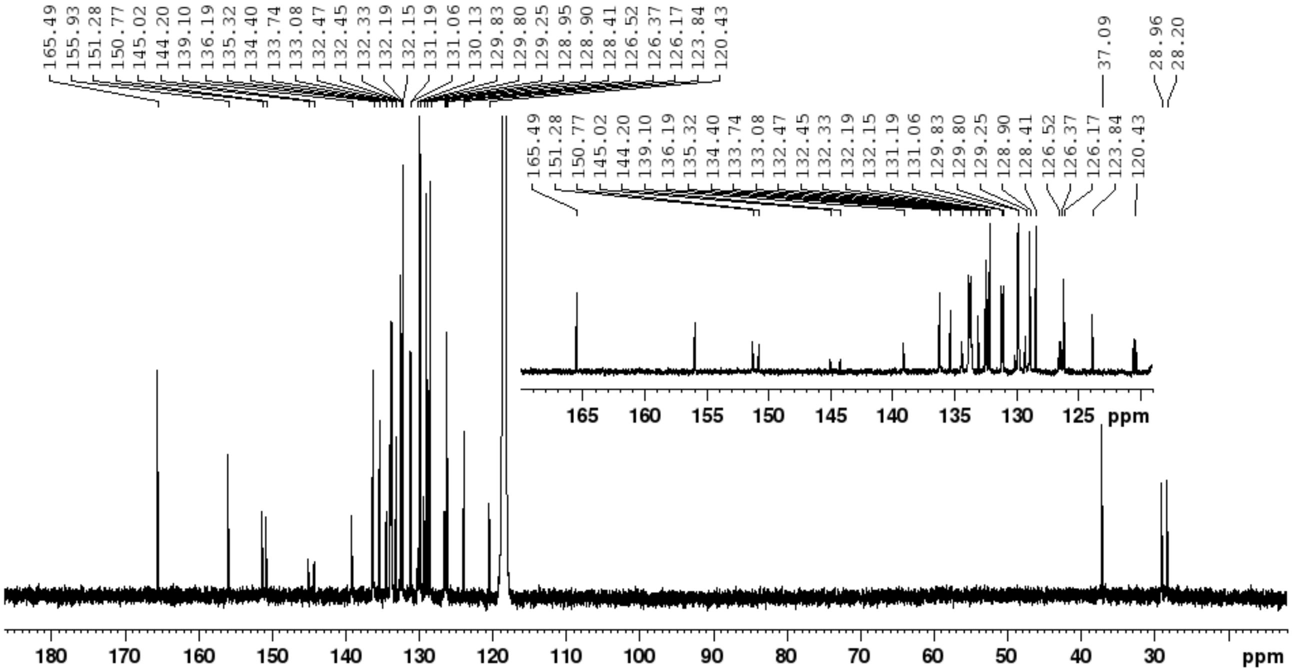

Supplementary Figure 5. ^{13}C NMR spectrum of **CuNIphen** (CD_3CN, 500 MHz).

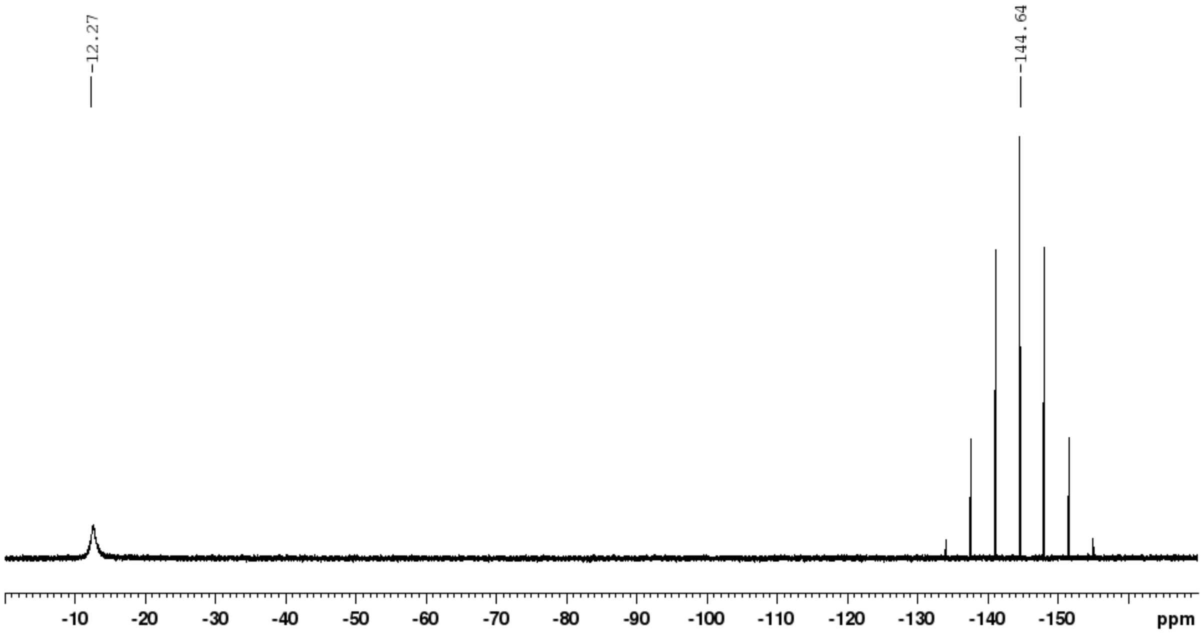

Supplementary Figure 6. ^{31}P NMR spectrum of **CuNIphen** (CD_3CN, 500 MHz).

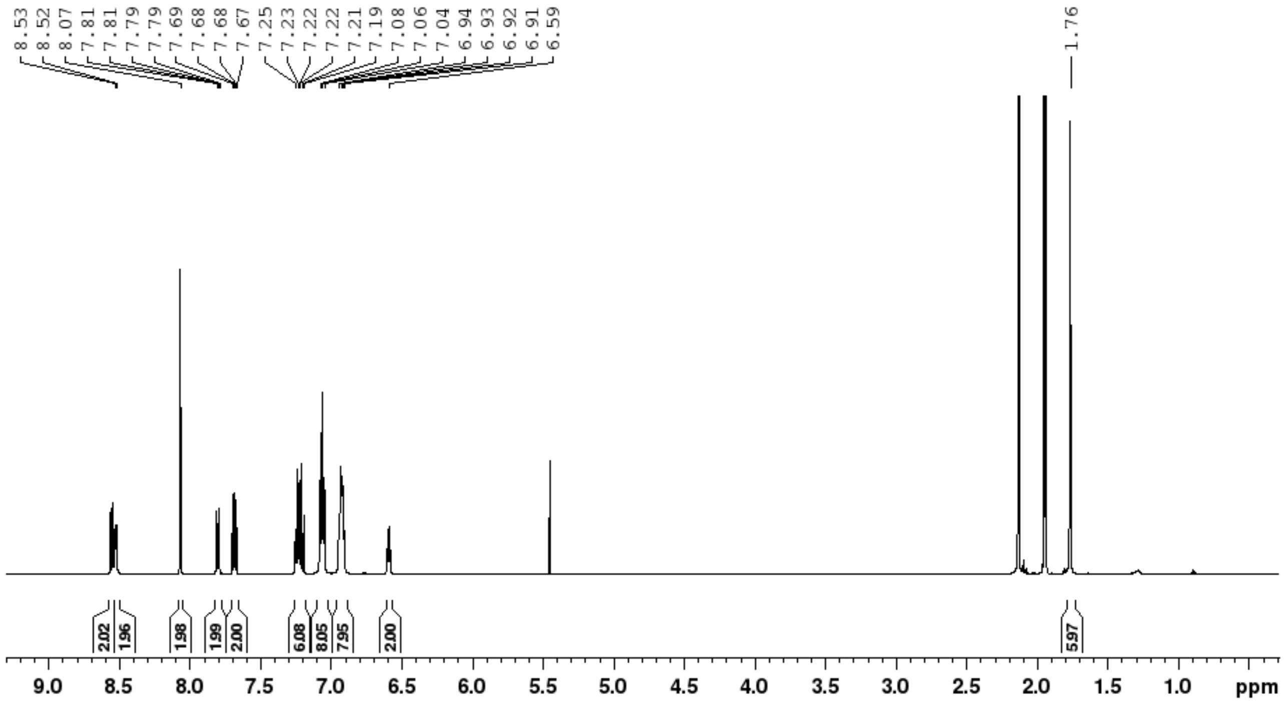

Supplementary Figure 7. ^{1}H NMR spectrum of **Cuphen** (CD_3CN, 500 MHz).

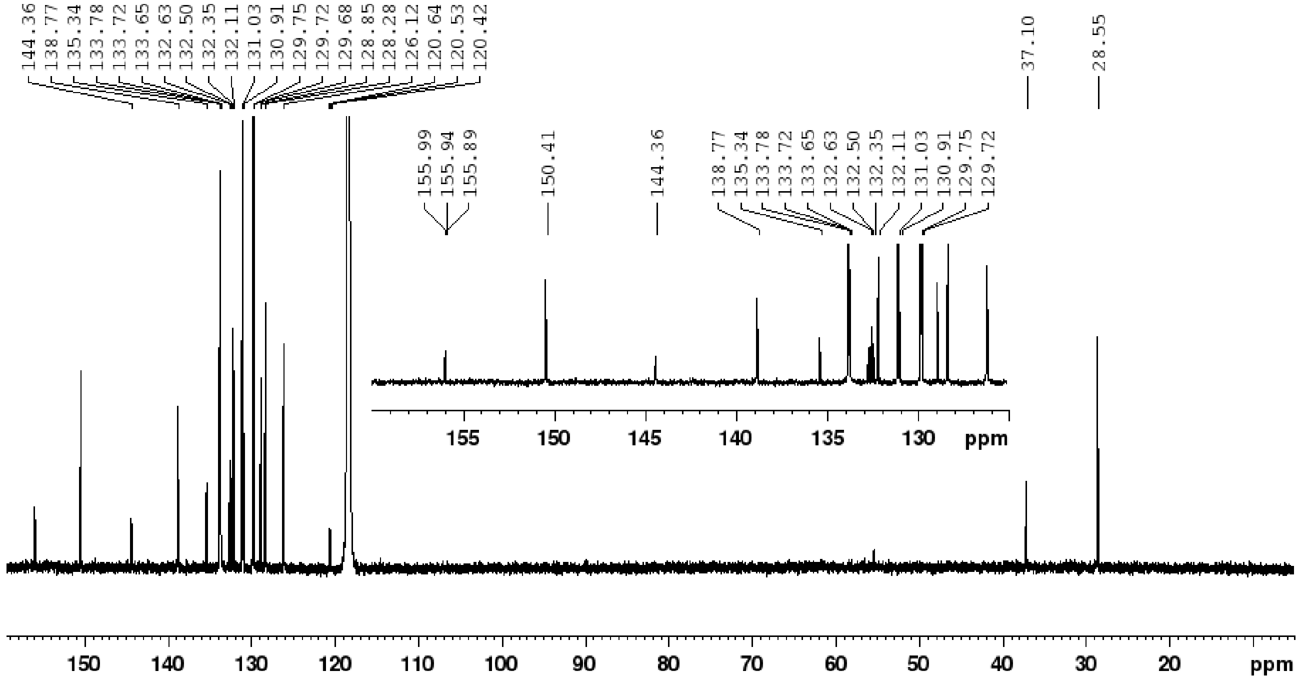

Supplementary Figure 8. ^{13}C NMR spectrum of **Cuphen** (CD_3CN, 500 MHz).

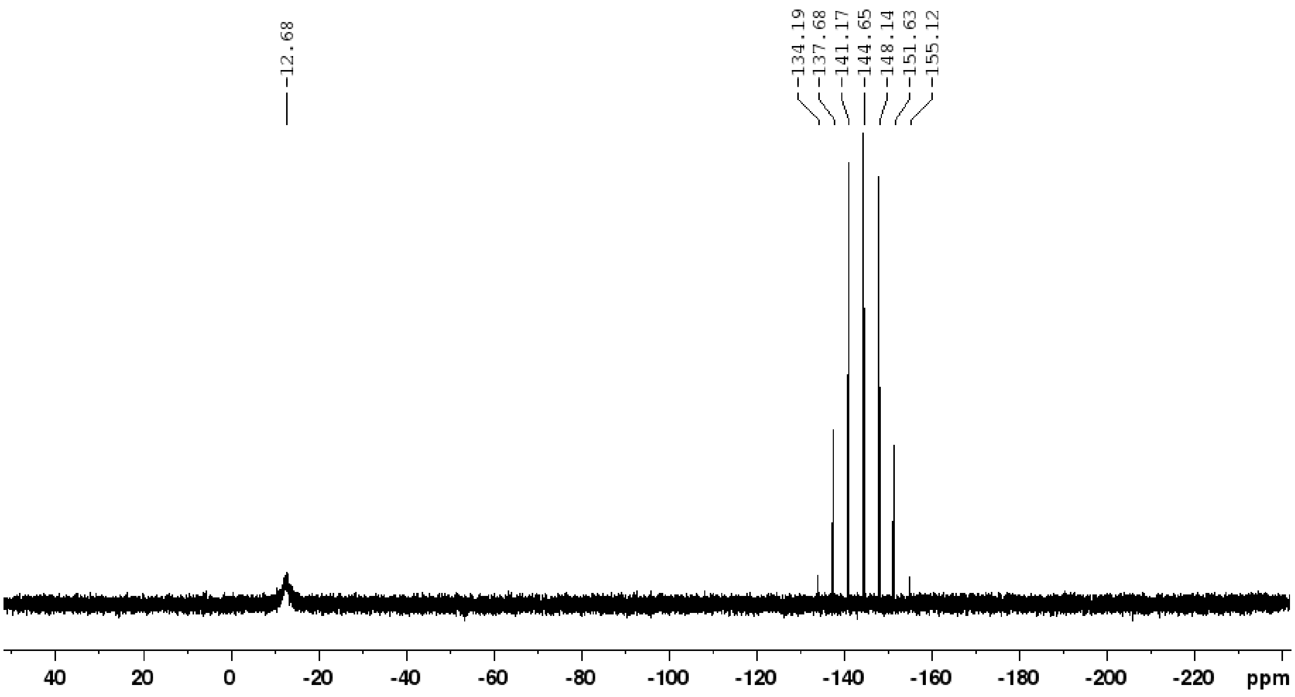

Supplementary Figure 9. ^{31}P NMR spectrum of **Cuphen** (CD_3CN, 500 MHz).

3.2 MS Spectra

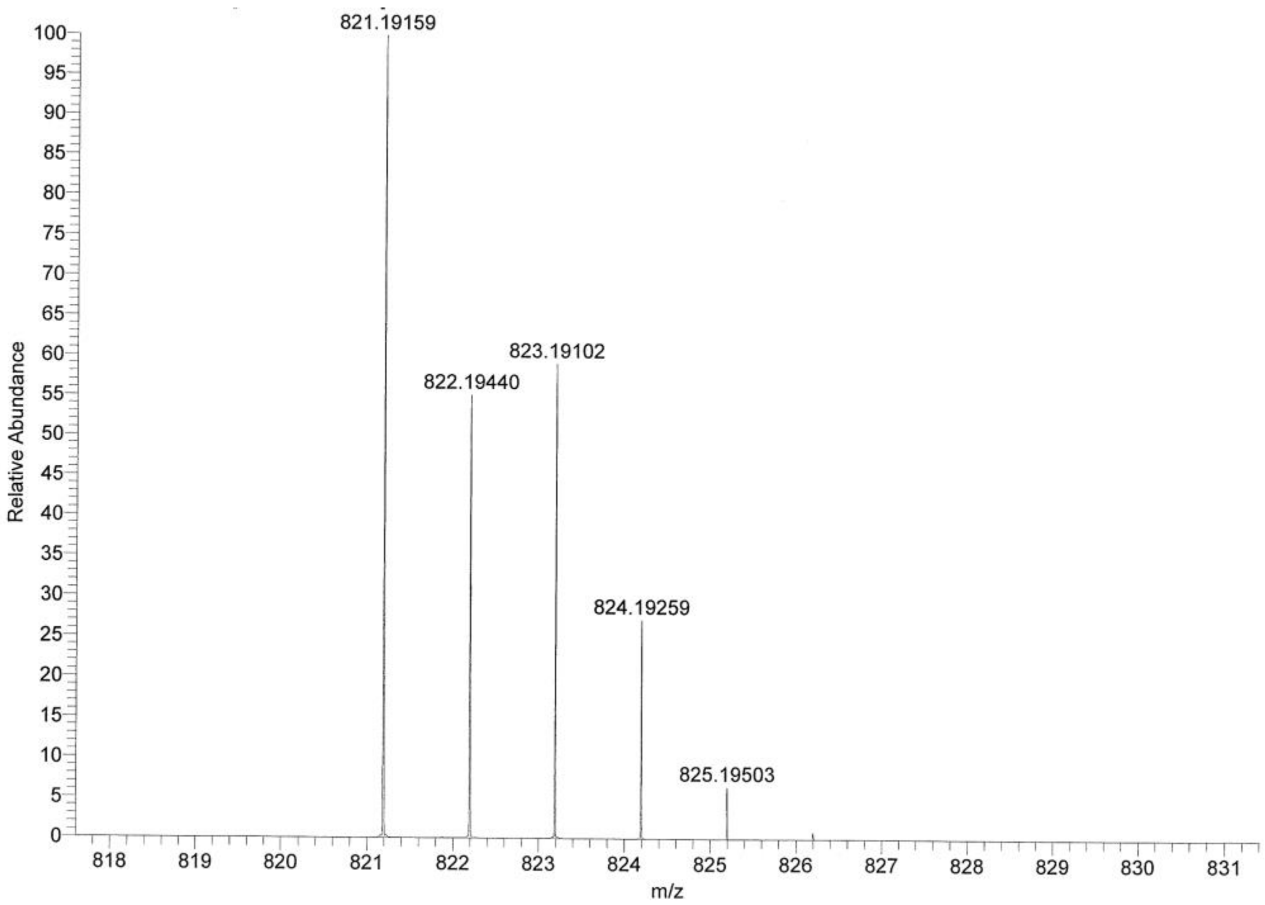

Supplementary Figure 10. Experimental ESI-MS of **Cuphen**. *m/z*: calcd. for $[C_{51}H_{40}CuN_2OP_2]^+$: 821.1906, found: 821.19159.

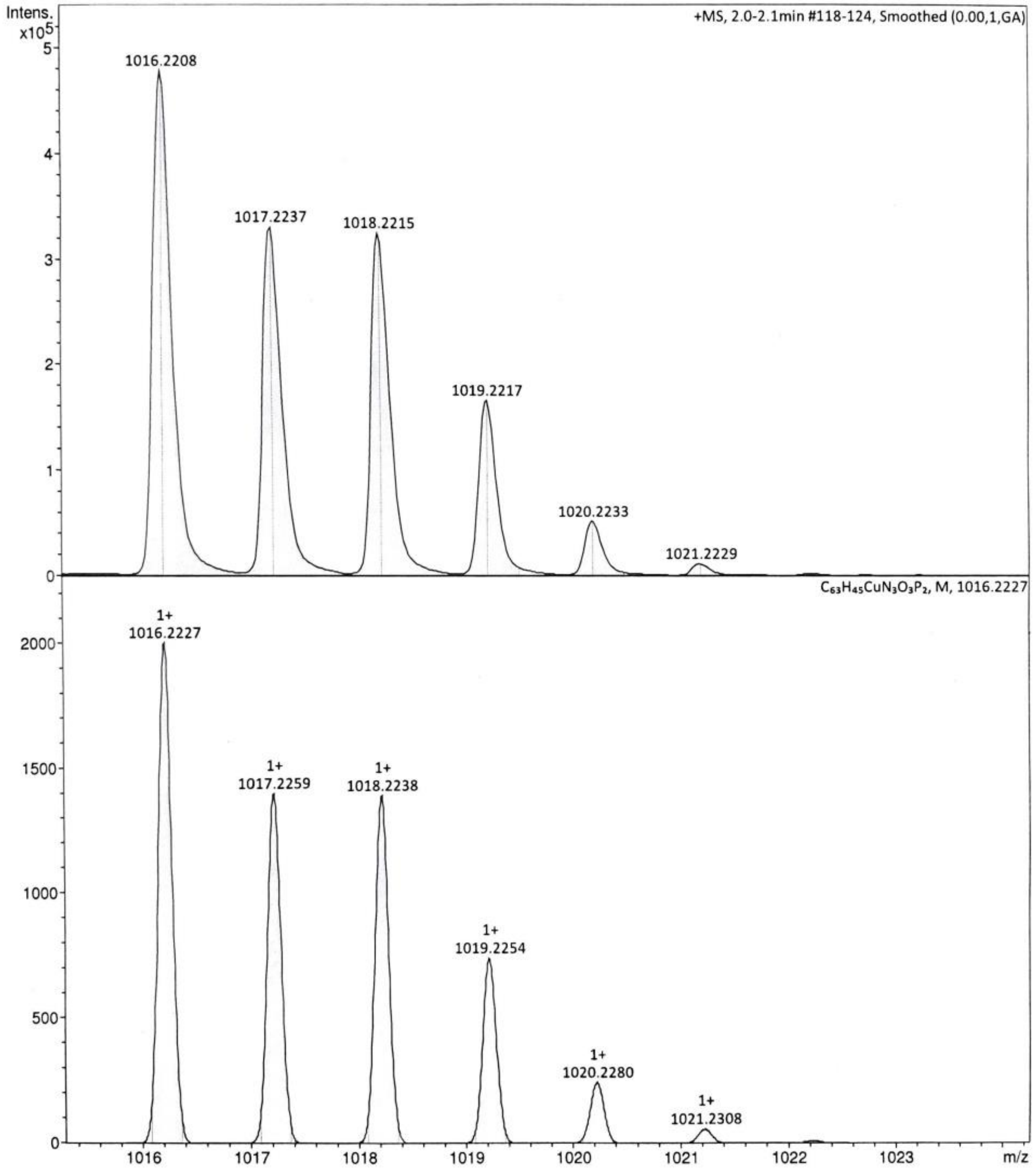

Supplementary Figure 11. Experimental (top) and simulated (bottom) ESI-MS of **CuNIphen**. *m/z* calcd. for $[C_{63}H_{45}CuN_3O_3P_2]^+$: 1016.2227, found: 1016.2208.

3.3 X-ray Analysis

Single crystals in suitable quality could be obtained by preparing a concentrated solution of the respective complex in dichloromethane and over layering with a thin film of ethanol and finally with a layer of *n*-heptane. Crystal growth at room temperature was completed in 4-7 days.

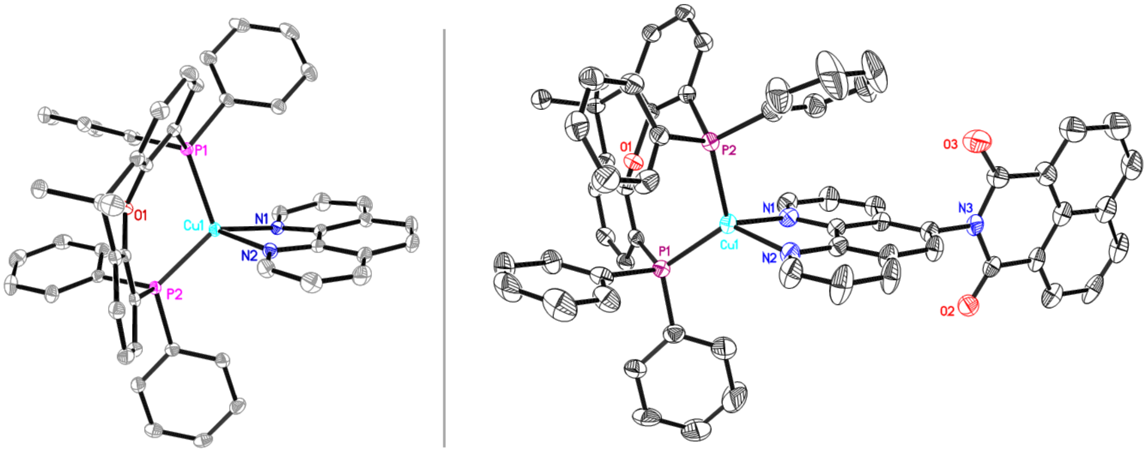

Supplementary Figure 12. Experimental solid-state structures of **Cuphen** (left) and **CuNIphen** (right) shown as ORTEP representation with thermal ellipsoids at a probability level of 50 %. Hydrogen atoms, solvent molecules and anions are omitted for clarity.

Supplementary Table 1. Selected bond lengths, bite angles and interplanar angles (N-Cu-N *vs*. P-Cu-P) of **Cuphen**. The table further compares the experimentally determined parameters (exp.) with those calculated theoretically with DFT (denoted italicized as *calc.*).

**Selected bond lengths**				**Selected angles**				
Atom 1	Atom 2	Length (pm): exp.	*vs. calc.*	Atom 1	Atom 2	Atom 3	Angle (°): exp.	*vs. calc.*
Cu	P1	228.49(5)	*229.5*	P1	Cu	P2	114.72(2)	*118.0*
Cu	P2	224.36(6)	*223.5*	P1	Cu	N1	106.64(4)	*101.8*
Cu	N1	205.1(1)	*210.7*	P1	Cu	N2	115.00(4)	*118.5*
Cu	N2	213.0(1)	*210.8*	P2	Cu	N1	129.87(4)	*128.8*
**Interplanar angle (°):** exp. *vs. calc.*				P2	Cu	N2	105.21(4)	*101.3*
	77.36	*87.63*		N1	Cu	N2	80.26(5)	*80.2*

Supplementary Table 2. Selected bond lengths, bite angles, interplanar angles (N-Cu-N *vs*. P-Cu-P) and substituent torsion angle (phen *vs*. naphthalimide) of **CuNIphen**. The bond length C*-N3 describes C-N bond from the phenanthroline to the naphthalimide. The table further compares the experimentally determined parameters (exp.) with those calculated theoretically with DFT (denoted italicized as *calc.*).

**Selected bond lengths**				**Selected angles**				
Atom 1	Atom 2	Length (pm): exp.	*vs. calc.*	Atom 1	Atom 2	Atom 3	Angle (°): exp.	*vs. calc.*
Cu	P1	224.7(2)	*223.8*	P1	Cu	P2	117.90(6)	*118.1*
Cu	P2	230.2(2)	*229.3*	P1	Cu	N1	112.5(1)	*114.9*
Cu	N1	209.8(5)	*212.1*	P1	Cu	N2	126.4(1)	*130.6*
Cu	N2	206.4(5)	*209.9*	P2	Cu	N1	110.3(1)	*102.4*
C*	N3	144.8(7)	*143.9*	P2	Cu	N2	103.2(1)	*102.2*
**Interplanar angle (°):** exp. *vs. calc.*				N1	Cu	N2	80.4(2)	*79.9*
	82.15	*86.70*						
**Substituent torsion angle (°):** exp. *vs. calc.*								
	88.97	*88.24*						

Supplementary Table 3. Crystal data and structure refinement for **Cuphen** (tsc6ck).

CCDC number	2165279
Sum formula	$C_{51}H_{40}CuF_6N_2OP_3$
Formula weight	967.30
Temperature	110(2) K
Wavelength	0.71073 Å
Crystal system, Space group	Monoclinic, $P2_1/n$ (no. 14)
Unit cell dimensions	a = 10.4815(3) Å; α = 90°.
	b = 21.4968(5) Å; β = 94.5320(10)°.
	c = 19.5726(3) Å; γ = 90°.
Volume, Z	4396.28(18) $Å^3$, 4
Density (calculated)	1.461 Mg/m^3
Absorption coefficient	0.673 mm^{-1}
F(000)	1984
Crystal size	0.610 x 0.380 x 0.250 mm^3
Completeness to θ = 25.242°	99.9%
Index ranges	-13 <= h <= 13, -28 <= k <= 28, -26 <= l <= 26
Reflections collected / indep. / obs.	117336 / 10895 (R_{int} = 0.0559) / 9067
Absorption correction	Gaussian
Max. and min. transmission	0.985 and 0.763
Refinement method	Full-matrix least-squares on F^2
Data / restraints / parameters	10895 / 0 / 579
Goodness-of-fit on F^2	1.037
Final R indices [$I>2\sigma(I)$]	R_1 = 0.0308, wR_2 = 0.0713
R indices (all data)	R_1 = 0.0421, wR_2 = 0.0768
Largest diff. peak and hole	0.347 and -0.378 e.$Å^{-3}$

Supplementary Table 4. Crystal data and structure refinement for **CuNIphen** (s2530lc_sq).

CCDC number	2165289
Empirical formula	$C_{63}H_{45}CuF_6N_3O_3P_3$
Formula weight	1162.47
Temperature	130(2) K
Wavelength	1.54178 Å
Crystal system, space group	Monoclinic, $P2_1/n$ (no. 14)
Unit cell dimensions	a = 11.5331(10) Å; α = 90°
	b = 32.835(3) Å; β =101.413(6)°
	c = 16.8772(14) Å; γ = 90°
Volume, Z	6264.8(10) $Å^3$, 4
Calculated density	1.232 Mg/m^3
Absorption coefficient	1.738 mm^{-1}
F(000)	2384
Crystal size	0.548 x 0.074 x 0.045 mm^3
Completeness to θ = 63.000°	96.5%
Limiting indices	13<=h<=10, -37<=k<=37, -14<=l<=19
Reflections collected / unique	40844 / 9785 [R_{int} = 0.0929]
Absorption correction	Semi-empirical from equivalents
Max. and min. transmission	0.7528 and 0.4487
Refinement method	Full-matrix least-squares on F^2
Data / restraints / parameters	9785 / 0 / 714
Goodness-of-fit on F^2	1.041
Final R indices [$I>2\sigma(I)$]	R_1 = 0.0834, wR_2 = 0.2021
R indices (all data)	R_1 = 0.1113, wR_2 = 0.2150
Largest diff. peak and hole	1.235 and -0.880 e.$Å^{-3}$

4 Cyclic voltammograms

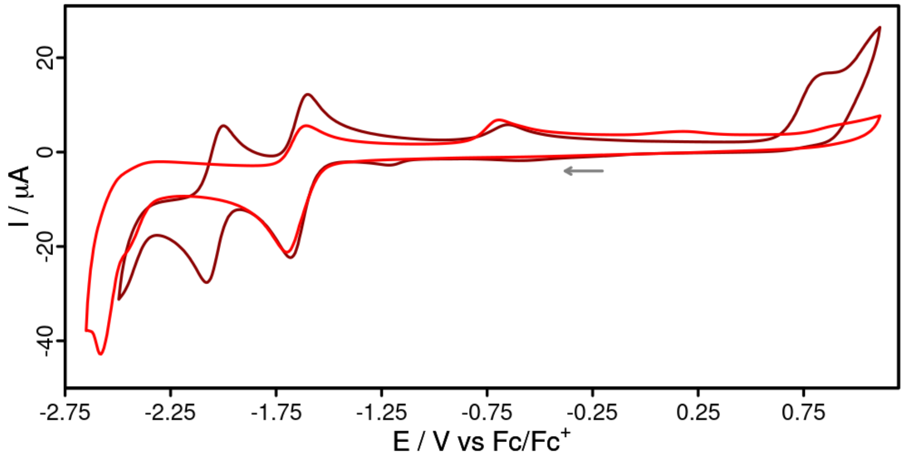

Supplementary Figure 13. Cyclic voltammograms of **NIphen** (red, 1 mM) and **CuNiPhen** (dark red, 1 mM) in acetonitrile solution referenced *vs.* the ferrocene/ferricenium (Fc/Fc^+) couple. Conditions: scan rate of 100 mVs^{-1}, $[Bu_4N][PF_6]$ (0.1 M) as supporting electrolyte.

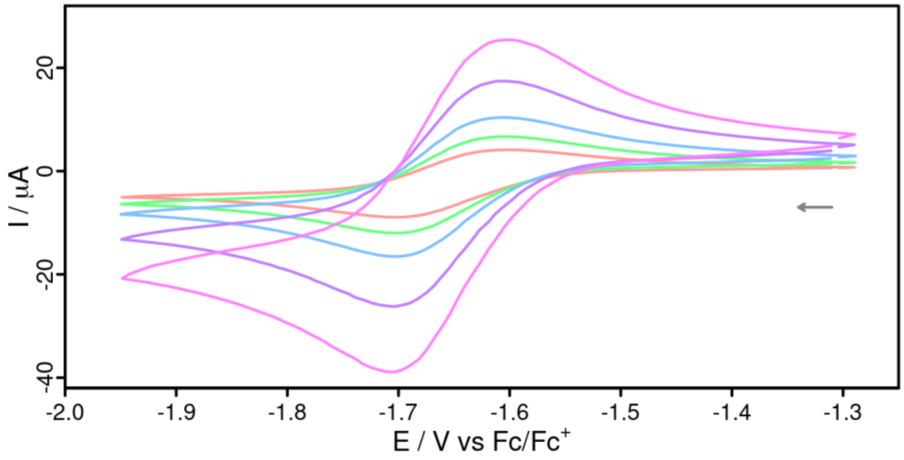

Supplementary Figure 14. Reductive events of the cyclic voltammograms of **NIphen** in acetonitrile solution referenced vs. the ferrocene/ferricenium (Fc/Fc^+) couple at different scan rates. Conditions: scan rate of 25 mVs^{-1} (light red), 50 mVs^{-1} (light green), 100 mVs^{-1} (light blue), 250 mVs^{-1} (light purple) and 500 mVs^{-1} (light magenta), with $[Bu_4N][PF_6]$ (0.1 M) as supporting electrolyte. The arrow illustrates the initial scan direction.

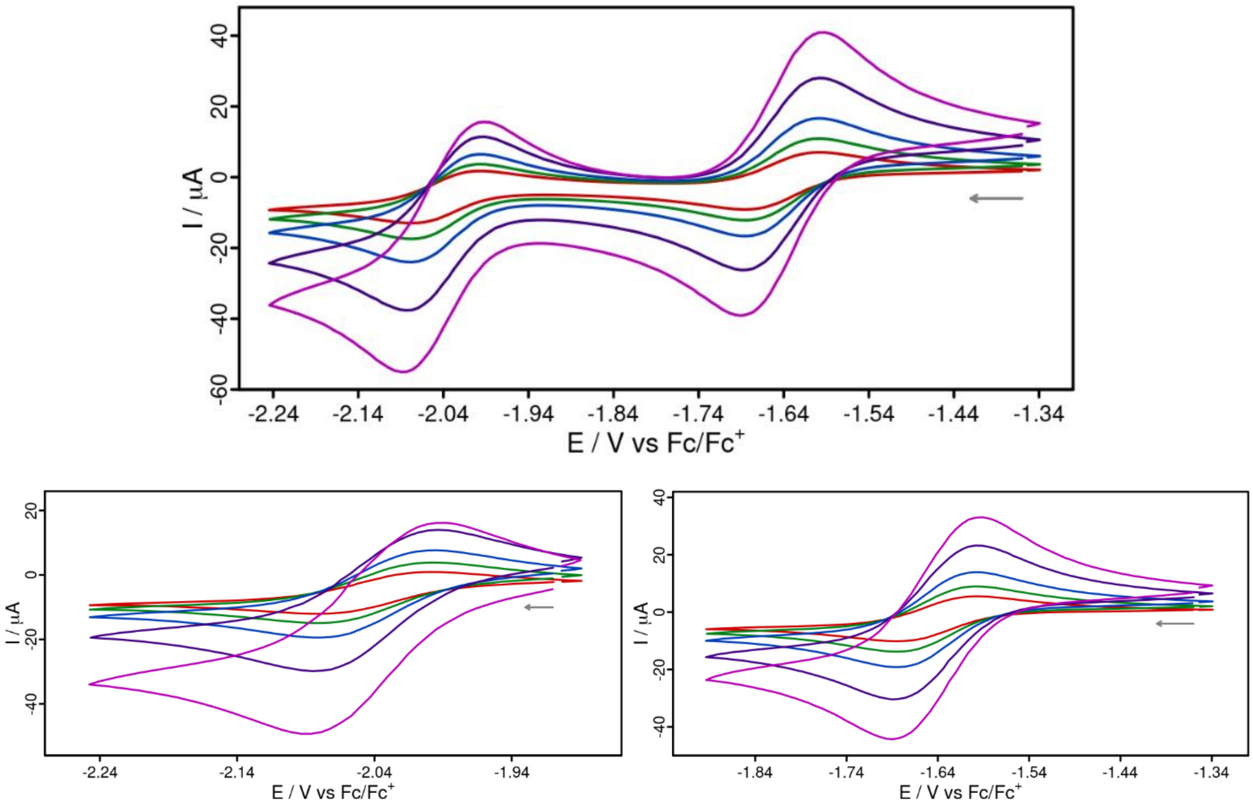

Supplementary Figure 15. Reductive events of the cyclic voltammograms of **CuNIPhen** in acetonitrile solution referenced vs. the ferrocene/ferricenium (Fc/Fc^+) couple at different scan rates. Conditions: scan rate of 25 mVs^{-1} (red), 50 mVs^{-1} (green), 100 mVs^{-1} (blue), 250 mVs^{-1} (purple) and 500 mVs^{-1} (magenta), with $[Bu_4N][PF_6]$ (0.1 M) as supporting electrolyte. The arrow illustrates the initial scan direction. Top: Both reduction events in one picture. Bottom left: the second reduction event. Bottom right: the first reduction event.

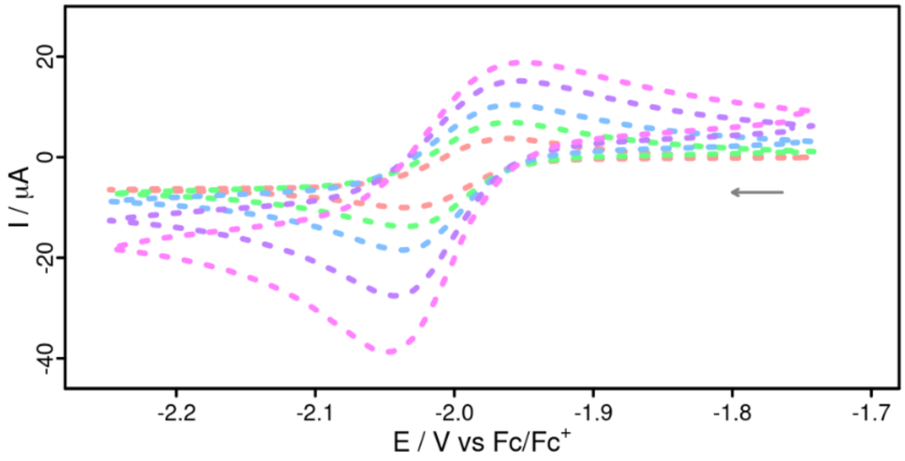

Supplementary Figure 16. Reductive events of the cyclic voltammograms of **Cuphen** in acetonitrile solution referenced vs. the ferrocene/ferricenium (Fc/Fc^+) couple at different scan rates. Conditions: scan rate of 25 mVs^{-1} (light red), 50 mVs^{-1} (light green), 100 mVs^{-1} (light blue), 250 mVs^{-1} (light purple) and 500 mVs^{-1} (light magenta), with $[Bu_4N][PF_6]$ (0.1 M) as supporting electrolyte. The arrow illustrates the initial scan direction.

5 Experimental UV/vis Absorption and Emission Spectra

5.1 Emission Spectra

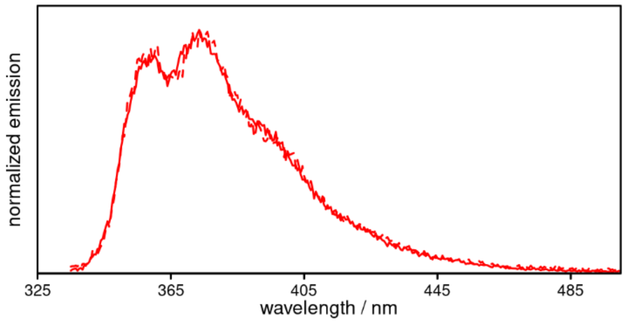

Supplementary Figure 17. Room temperature emission spectra of **NIphen** (red) in aerated acetonitrile (solid) and in aerated dichloromethane (dashed) with λ_{ex} = 325 nm.

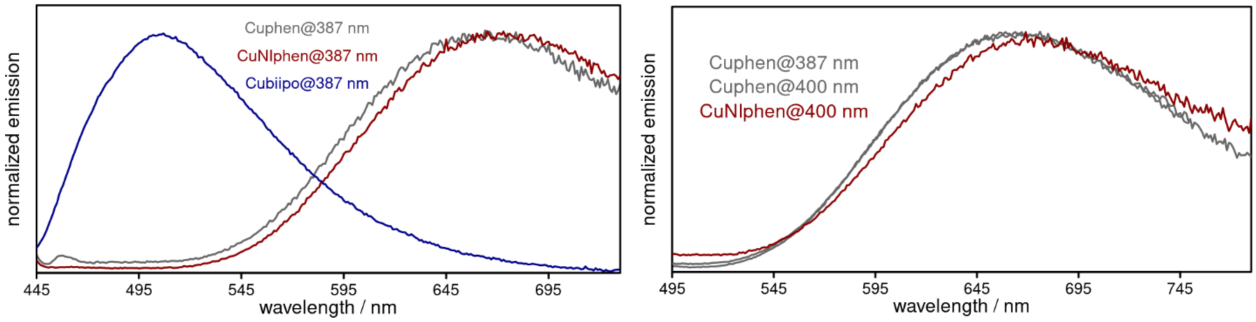

Supplementary Figure 18. Room temperature emission spectra of **CuNIphen** (dark red), **Cuphen** (grey) and **Cubiipo** (dark blue) recorded in aerated dichloromethane (left) and degassed dichloromethane (right) with λ_{ex} = 387 nm and λ_{ex} = 400 nm.

5.2 Photostability Measurements of CuNIphen

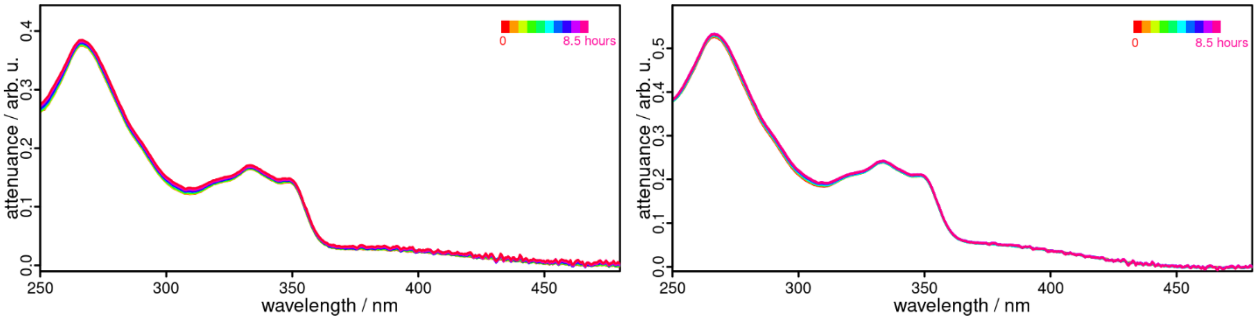

Supplementary Figure 19. UV/vis absorption photostability measurements of **CuNIphen** in oxygen free acetonitrile in the dark (left) and under irradiation (right) with 150 W Xe arc lamp using a 0.5 OD filter for 8.5 hours.

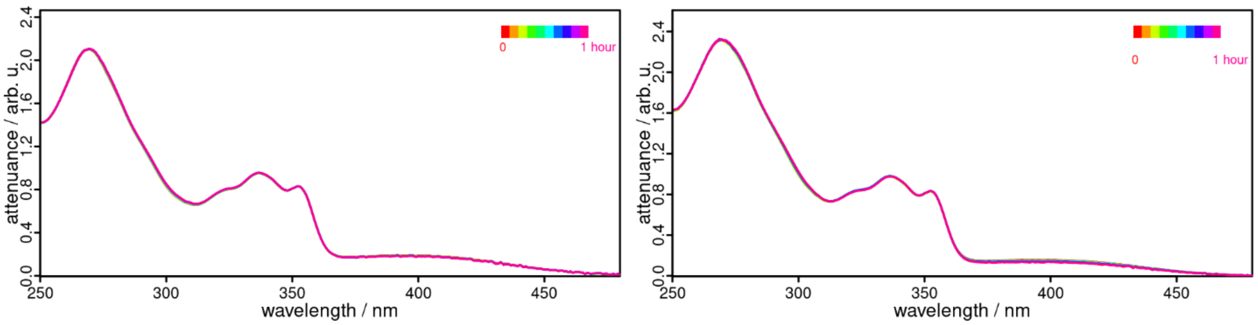

Supplementary Figure 20. UV/vis absorption photostability measurements of **CuNIphen** in aerated dichloromethane in the dark (left) and under irradiation (right) with 150 W Xe arc lamp using a 0.5 OD filter and a 360 nm long pass filter for 1 hour.

5.3 Singlet Oxygen Detection

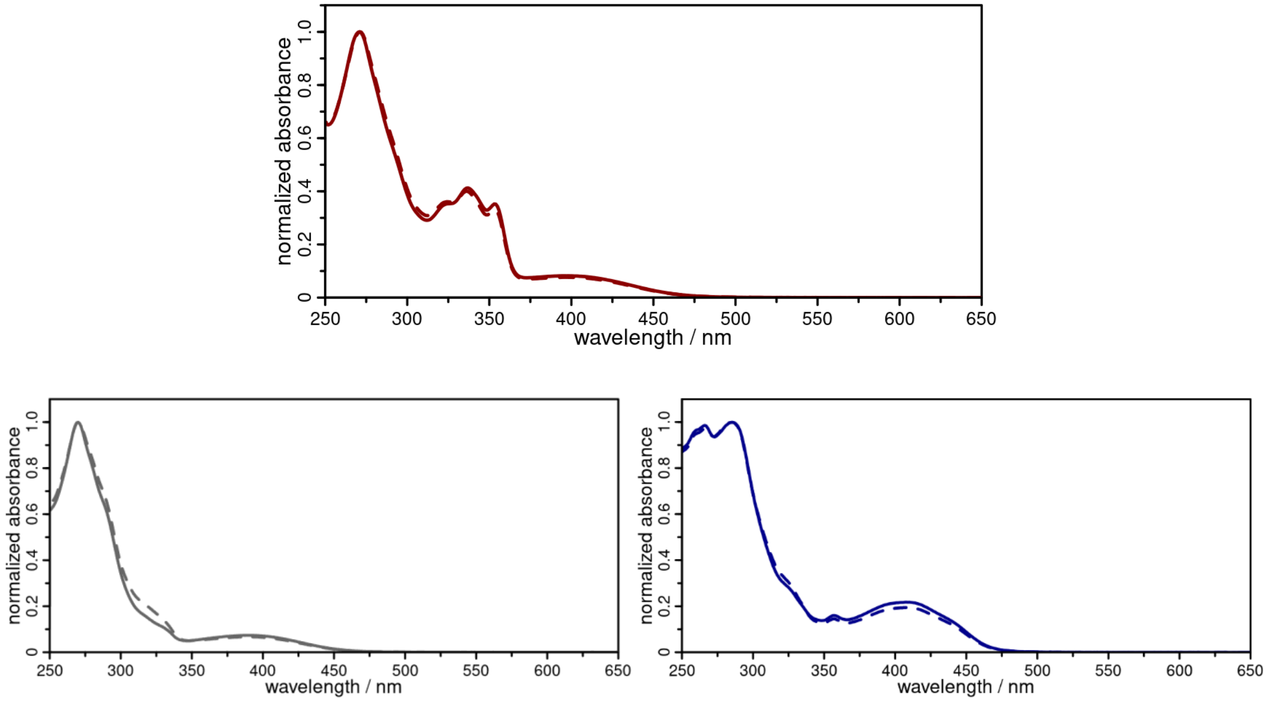

Supplementary Figure 21. UV/vis spectra of **CuNIphen** (dark red), **Cuphen** (grey) and **Cubiipo** (dark blue) before (solid) and after (dashed) $^1O_2$ measurement excited at 387 nm in dichloromethane with OD at 387 nm around 0.1.

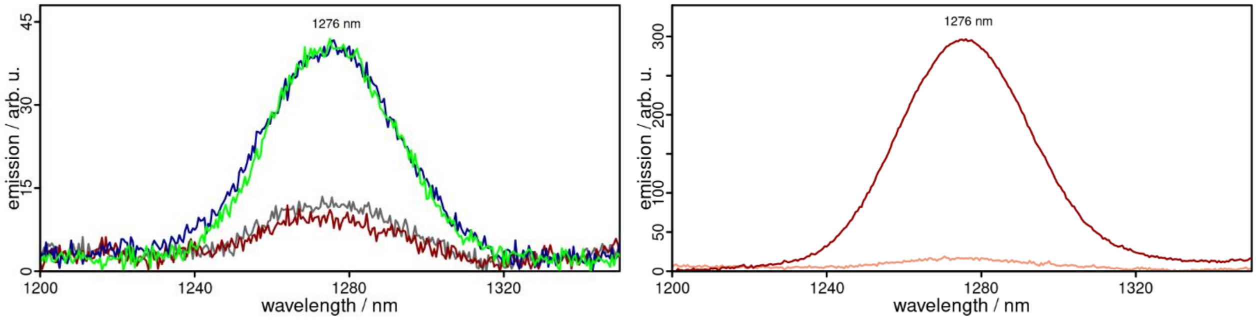

Supplementary Figure 22. Left: $^1O_2$ emission spectra after baseline correlation of phenalenone (green), **CuNIphen** (dark red), **Cuphen** (grey) and **Cubiipo** (dark blue) in dichloromethane upon excitation at 387 nm with OD at 387 nm around 0.1. Right: $^1O_2$ emission spectra **CuNIphen** in deuterated dichloromethane (dark red) and dichloromethane (light red) upon excitation at 387 nm with OD at 387 nm around 0.18.

6 Transient Absorption Spectra

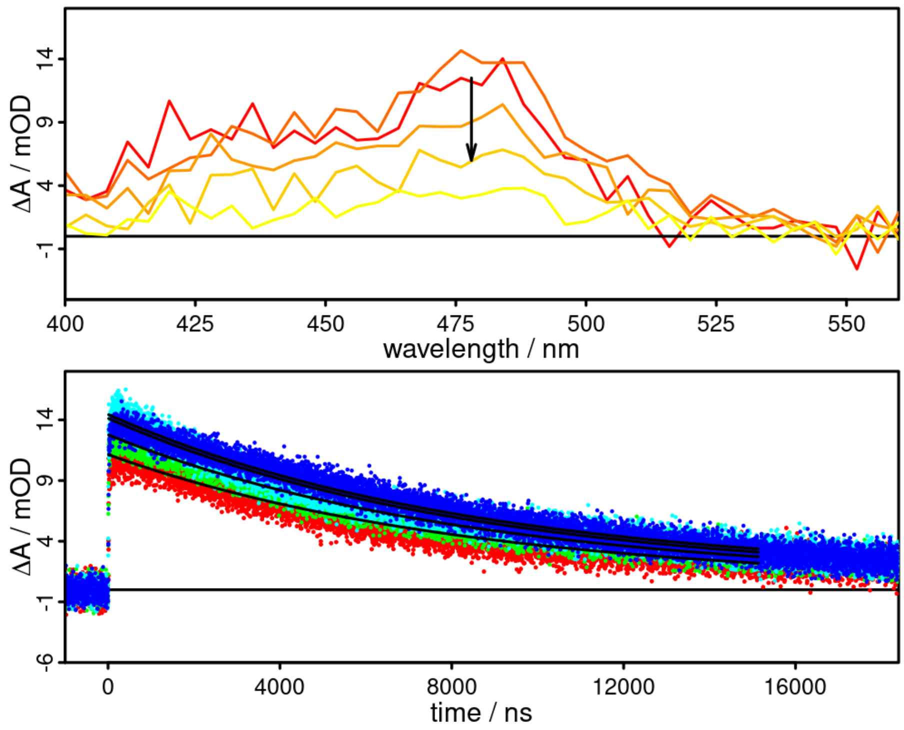

Supplementary Figure 23. Transient absorption spectra (top) and respective kinetics (bottom) of **NIphen** in acetonitrile excited at 355 nm under inert conditions. Top: Spectra from 50 (red) to 15000 ns (yellow). Bottom: Kinetics at 464 (red), 468 (green), 476 (cyan) and 480 nm (blue), the black line belongs to the fits with a time constant of 8.4 µs.

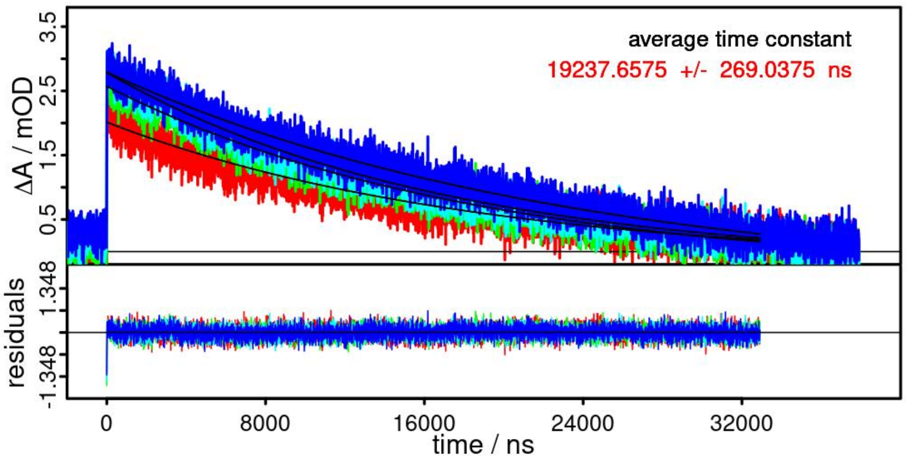

Supplementary Figure 24. Single wavelength kinetic analysis at 462 (red), 468 (green), 474 (cyan) and 480 nm (blue) of **CuNIphen** in acetonitrile excited at 355 nm under inert conditions. The black line belongs to the fits with an average time constant of 19.24 ± 0.27 µs.

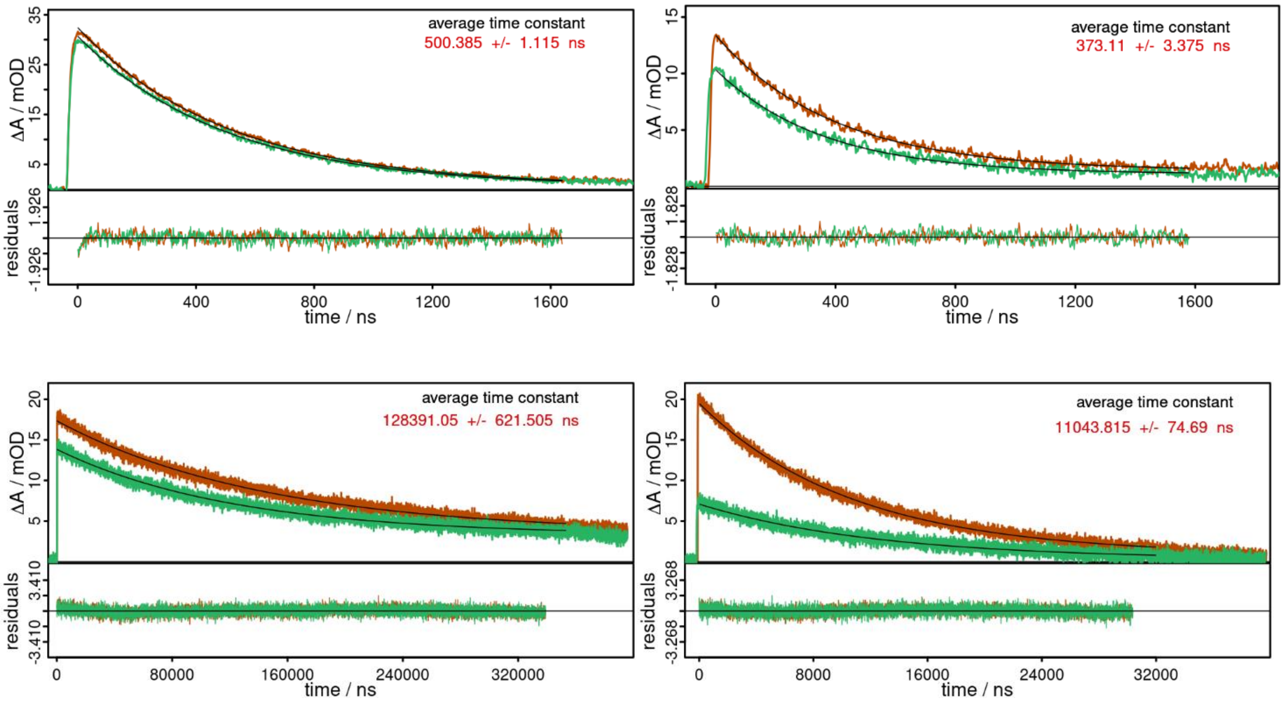

Supplementary Figure 25. Single wavelength kinetic analysis at 480 (green) and 510 nm (dark orange) of **Cuphen** (top left), **CuNIphen** (top right), **Cubiipo** (bottom left), and **NIphen** (bottom right) in deaerated dichloromethane excited at 355 nm. The black line belongs to the fits.

7 Calculated Ground State Structures

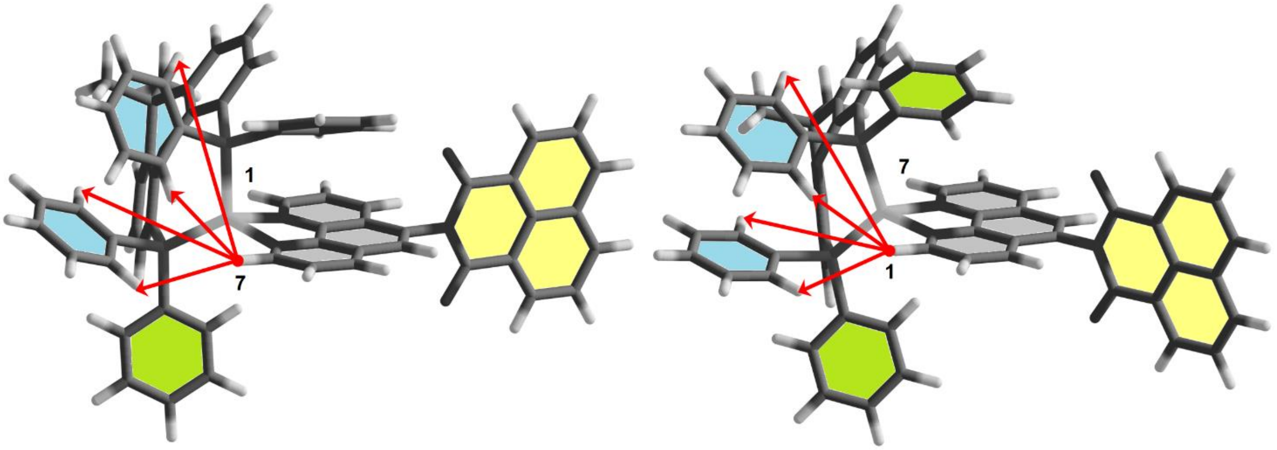

Supplementary Figure 26. Calculated ground state structures simulated in MeCN (CPMC) obtained from DFT (B3LYP-D3(BJ)/def2-TZVP) of the two possible regioisomers of **CuNIphen**. The thermodynamic energy difference between both isomers is predicted to be 1.39 kJ/mol (left: energetically favored). Additionally the graphic illustrates possible through-space interaction concerning H-H NOESY: The red arrows indicate mutual interaction between the *o*-protons of the phenyl rings (blue) and the proton 7 (left structure, 9-position) or the proton 1 (right structure, 2-position).

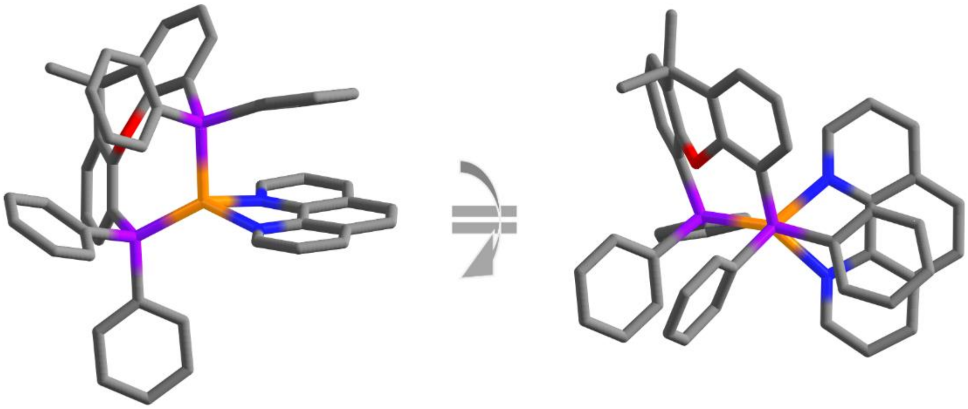

Supplementary Figure 27. Calculated ground state structures simulated in MeCN (CPMC) obtained from DFT (B3LYP-D3(BJ)/def2-TZVP) of **Cuphen** depicted as side-view (left) and top-view (right).

8 Calculated UV/vis Spectra, Differential Density Plots and Spin Densities

8.1 Cuphen

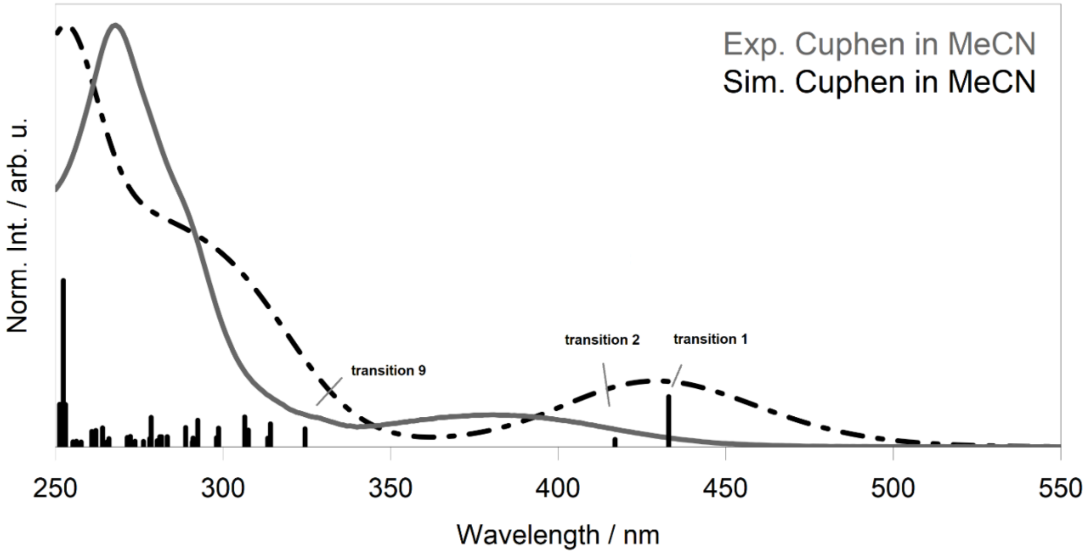

Supplementary Figure 28. Calculated (black, dashed) and experimental (gray) UV/vis spectrum of **Cuphen**.

Supplementary Table 5. Excitation energies, transition moments and corresponding transitions of **Cuphen** obtained from TD-DFT simulated in MeCN. Note that only excitations with an oscillator strength > 0.01 and corresponding orbital contributions with an |coeff.|2 *100 ≥ 0.1 are shown.

State	Excitation energy		Oscillator	Dominant contribution			Transition
#	cm^{-1}	nm	strength	$\|coeff.\|^2$*100	occ. orb.	virt. orb.	
1	23094	433	0.1191	0.9518	HOMO	LUMO	$d_{Cu} \rightarrow \pi^*_{phen}$
2	23979	417	0.0177	0.9720	HOMO	LUMO+1	$d_{Cu} \rightarrow \pi^*_{phen}$
9	30819	325	0.0429	0.9593	HOMO	LUMO+3	$d_{Cu} \rightarrow \pi^*_{phen}$

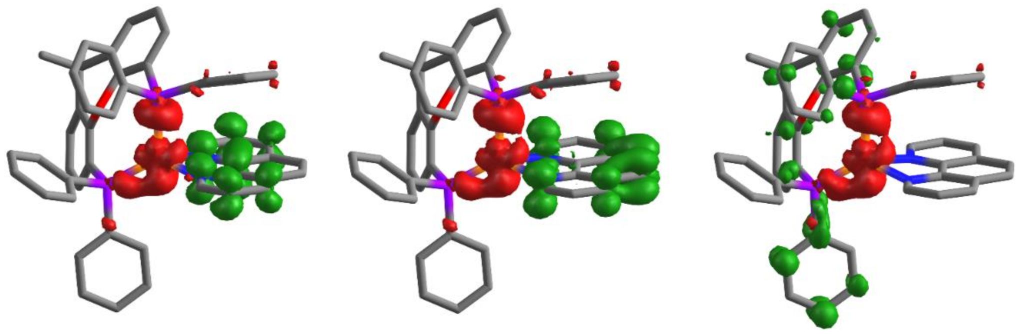

Supplementary Figure 29. Difference density plots of selected transitions calculated for **Cuphen** (B3LYP-D3(BJ)/def2-TZVP). Transitions from left to right: 1,2 and 9 (all assigned as MLCTs). Isosurface value: 0.003.

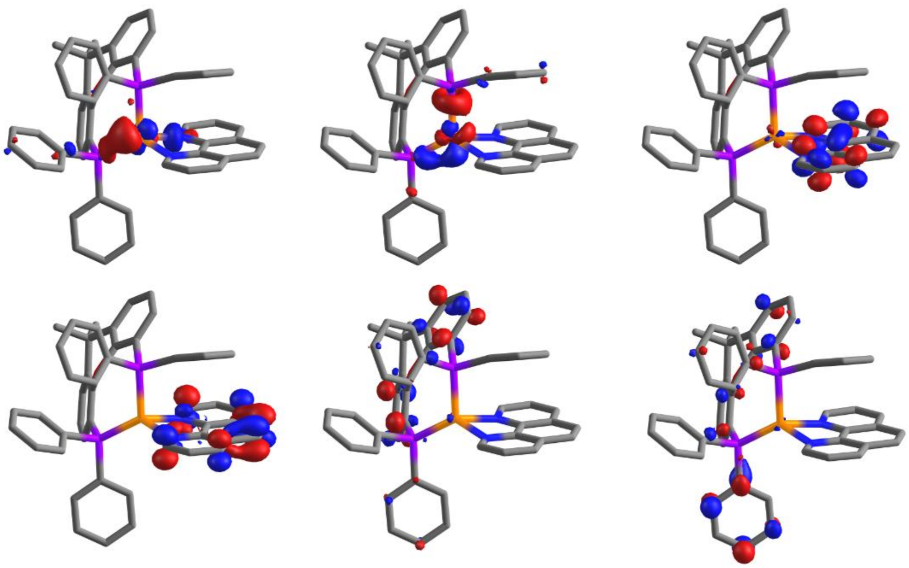

Supplementary Figure 30. Representation of selected orbitals of **Cuphen** (B3LYP-D3(BJ)/def2-TZVP). Top (left to right): HOMO-1, HOMO, LUMO. Bottom (left to right): LUMO+1, LUMO+2, LUMO+3. Isosurface value: 0.06.

8.2 CuNIphen

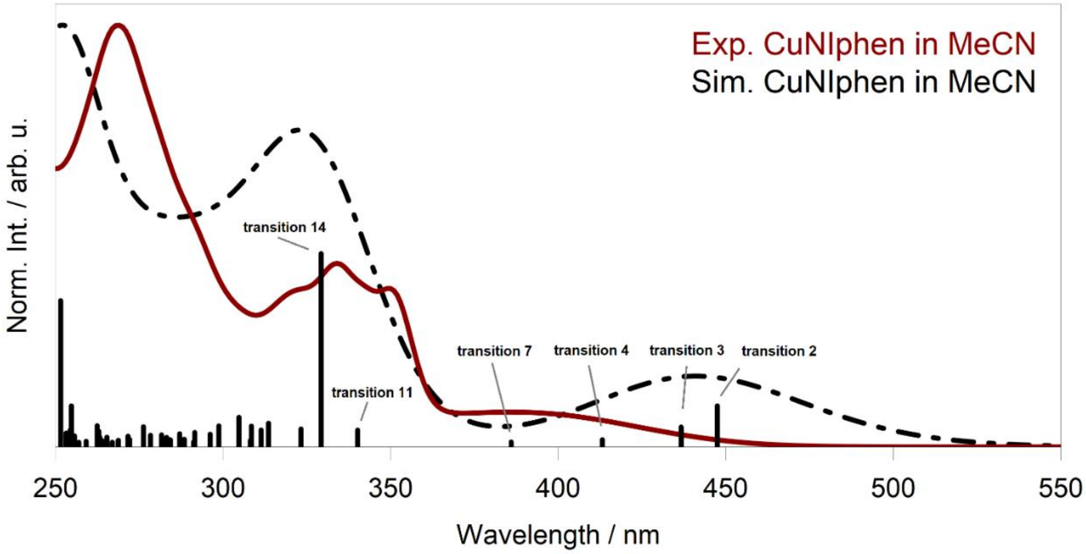

Supplementary Figure 31. Calculated (black, dashed) and experimental (red) UV/vis spectrum of **CuNIphen**.

Supplementary Table 6. Excitation energies, transition moments and corresponding transitions of **CuNIphen** obtained from TD-DFT simulated in MeCN. Note that only excitations with an oscillator strength > 0.01 and corresponding orbital contributions with an |coeff.|2 *100 ≥ 0.1 are shown.

State	Excitation energy		Oscillator	Dominant contribution			Transition
#	cm^{-1}	nm	strength	|coeff.|²*100	occ. orb.	virt. orb.	
2	22346	448	0.0974	0.9542	HOMO	LUMO+1	$d_{Cu} \rightarrow \pi^*_{phen}$
3	22897	437	0.0469	0.9577	HOMO	LUMO+2	$d_{Cu} \rightarrow \pi^*_{phen}$
4	24200	413	0.0169	0.1240	HOMO-2	LUMO+1	$d_{Cu} \rightarrow \pi^*_{phen}$
				0.6653	HOMO-1	LUMO+1	$d_{Cu} \rightarrow \pi^*_{phen}$
7	25904	386	0.0115	0.1995	HOMO-2	LUMO	$d_{Cu} \rightarrow \pi^*_{NI}$
				0.5165	HOMO-2	LUMO+1	$d_{Cu} \rightarrow \pi^*_{phen}$
				0.1685	HOMO-1	LUMO+2	$d_{Cu} \rightarrow \pi^*_{phen}$
11	29398	340	0.0397	0.7174	HOMO-5	LUMO	$d_{Cu}, \pi_{phen}, \pi_{xant} \rightarrow \pi^*_{NI}$
				0.1711	HOMO-4	LUMO	$\pi_{NI} \rightarrow \pi^*_{NI}$
14	30363	329	0.4581	0.1635	HOMO-5	LUMO	$d_{Cu}, \pi_{phen}, \pi_{xant} \rightarrow \pi^*_{NI}$
				0.7540	HOMO-4	LUMO	$\pi_{NI} \rightarrow \pi^*_{NI}$

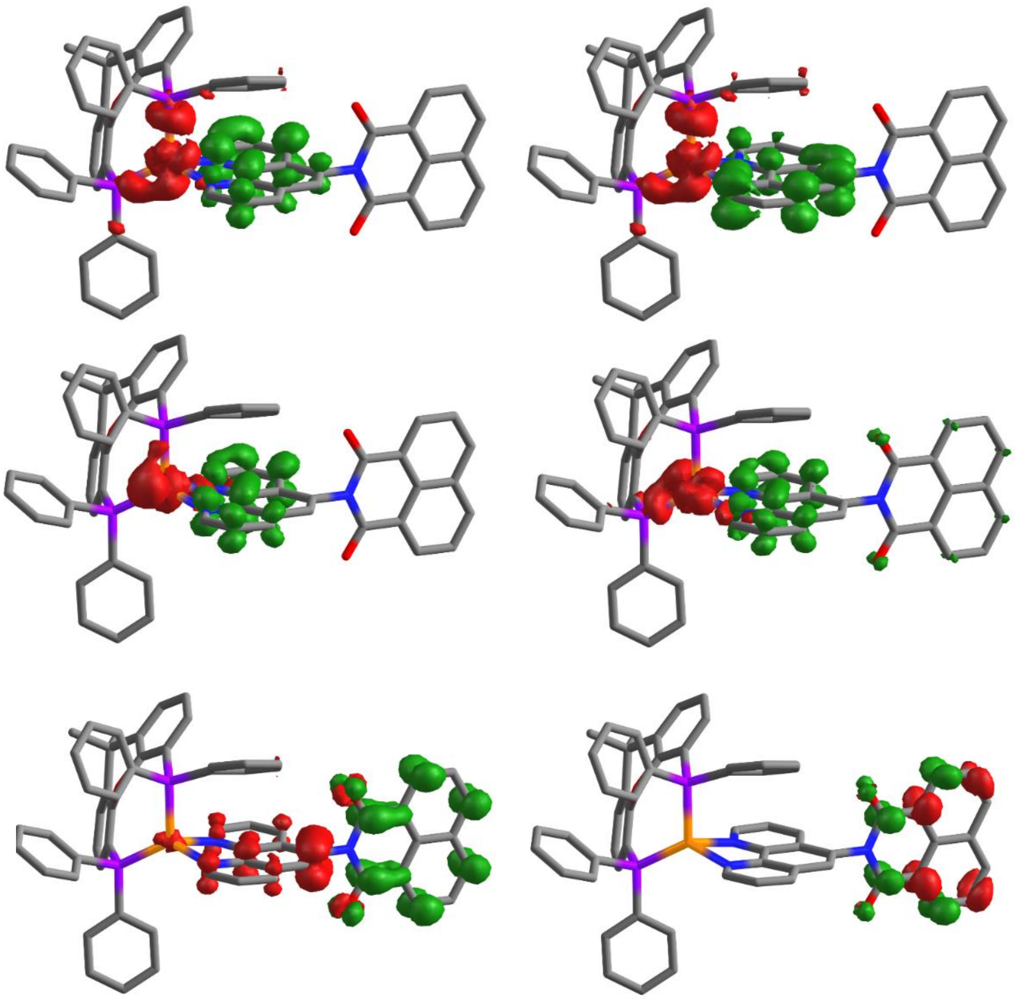

Supplementary Figure 32. Difference density plots of selected transitions calculated for **CuNIphen** (B3LYP-D3(BJ)/def2-TZVP). Transitions from left to right: **top**: 2 and 3 (both assigned as MLCT); **center**: 4 and 7 (both assigned as MLCT); **bottom**: 11 (left, assigned as ILCT with partial MLCT character) and 14 (right, assigned as mainly LC). Isosurface value: 0.003.

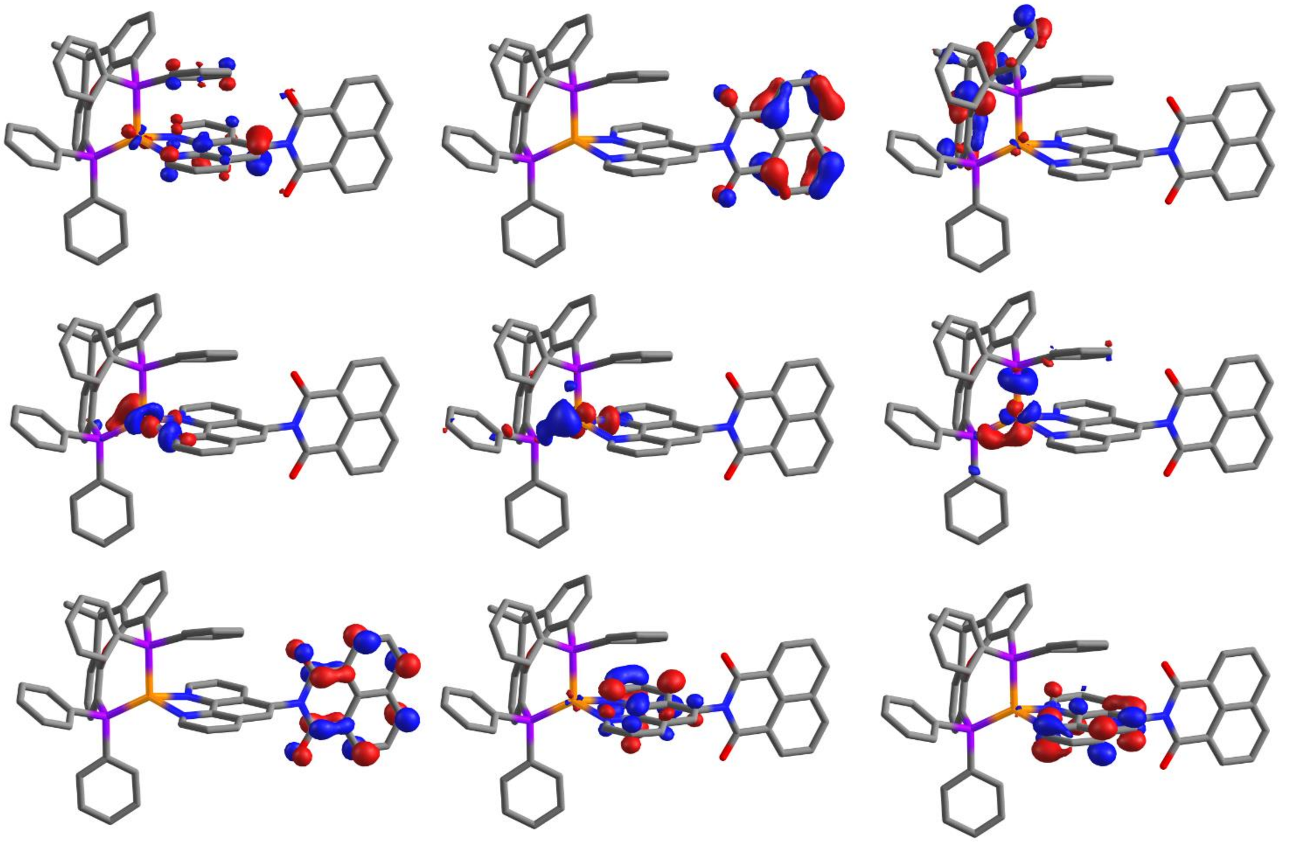

Supplementary Figure 33. Representation of selected orbitals of **CuNIphen** (B3LYP-D3(BJ)/def2-TZVP). Top (left to right): HOMO-5, HOMO-4, HOMO-3. Middle (left to right): HOMO-2, HOMO-1, HOMO. Bottom (left to right): LUMO, LUMO+1, LUMO+2. Isosurface value: 0.06.

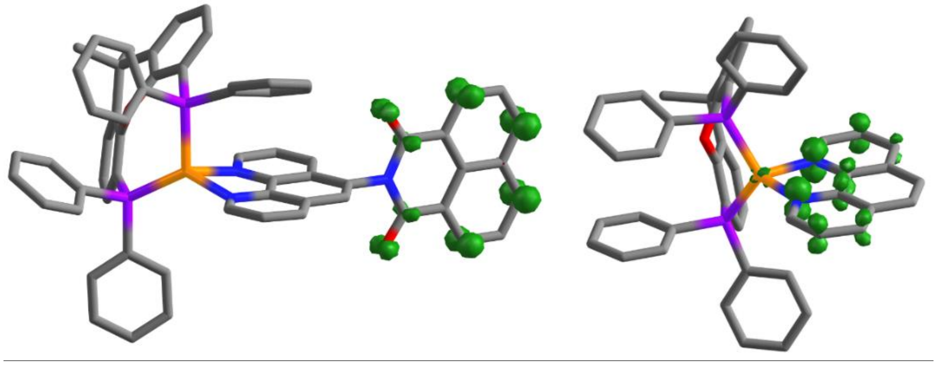

Supplementary Figure 34. Calculated spin density of the optimized singly reduced species of **CuNIphen** (left) and **Cuphen** (right) simulated in MeCN (B3LYP-D3(BJ)/def2-TZVP). Isosurface value: 0.01.

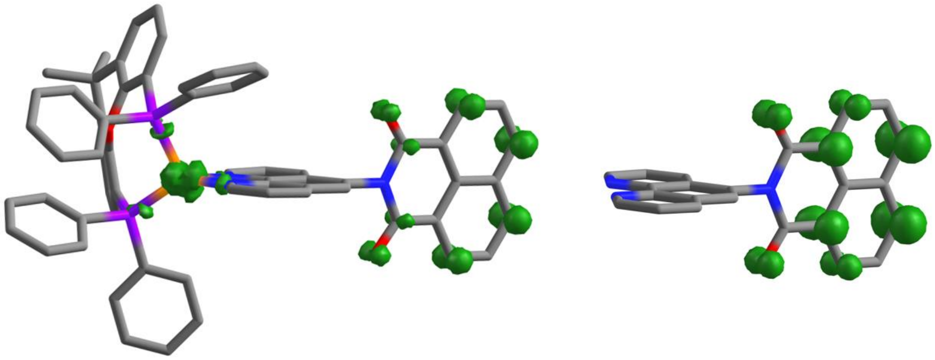

Supplementary Figure 35. Calculated spin density of the optimized lowest triplet state of **CuNIphen** (left) and **NIphen** (right) simulated in MeCN (B3LYP-D3(BJ)/def2-TZVP, CPMC). Please note that identical calculations applying the two solvents CH_2Cl_2 (see main text) and MeCN each yielded almost the same results. Isosurface value: 0.01.
